# Efficacy of Chinese herbal medicine in patients with osteoporosis: a systematic review and meta-analysis

**DOI:** 10.3389/fmed.2025.1620264

**Published:** 2025-07-25

**Authors:** Jin Wang, Fei Teng

**Affiliations:** ^1^Department of Traditional Chinese Medicine, The People’s Hospital of Pizhou, Pizhou, China; ^2^Department of Orthopedic, The People’s Hospital of Pizhou, Pizhou, China

**Keywords:** Chinese herbal medicine, osteoporosis, systematic review, meta-analysis, bone mineral density

## Abstract

**Introduction:**

Current evidence from randomized controlled trials (RCTs) supports the anti-osteoporotic properties of Chinese Herbal Medicine (CHM); however, its therapeutic advantages over conventional treatments remain inconclusive. This study aimed to compare the therapeutic effects of CHM with those of conventional therapy in patients with osteoporosis, using a meta-analysis approach.

**Methods:**

A systematic search of PubMed, Embase, Cochrane Library, CNKI, and Wanfang databases was conducted through March 2025 to identify eligible RCTs. The weighted mean difference (WMD) and 95% confidence intervals (CI) were used as effect estimates, with pooled analyses calculated using a random-effects model. Additional exploratory analyses included sensitivity and subgroup analyses.

**Results:**

Eighteen RCTs involving a total of 1,816 patients with osteoporosis were included in the meta-analysis. CHM was associated with increased bone mineral density (BMD) at the lumbar spine (WMD: 0.09; 95% CI: 0.04 to 0.13; *p* < 0.001), femoral neck (WMD: 0.09; 95% CI: 0.02 to 0.17; *p* = 0.015), and Ward’s triangle area (WMD: 0.08; 95% CI: 0.01 to 0.15; *p* = 0.025). However, CHM showed no significant effect on BMD at the greater trochanter of the femur (WMD: 0.01; 95% CI: −0.03 to 0.05; *p* = 0.698). Additionally, CHM was not associated with changes in alkaline phosphatase (WMD: 0.98; 95% CI: −6.88 to 8.83; *p* = 0.808), serum calcium (WMD: 0.08; 95% CI: −0.09 to 0.25; *p* = 0.372), or serum phosphorus (WMD: -0.05; 95% CI: −0.22 to 0.12; *p* = 0.574).

**Conclusion:**

Chinese Herbal Medicine was associated with significant improvements in BMD at the lumbar spine, femoral neck, and Ward’s triangle area compared to conventional therapies, though the evidence is limited by moderate study quality and high heterogeneity. The findings suggest potential benefits of CHM in specific skeletal sites, but further rigorous trials are needed to confirm efficacy.

**Systematic review registration:**

INPLASY platform (number: INPLASY202530115).

## Introduction

Osteoporosis is a systemic skeletal disorder characterized by bone loss and deterioration of bone microarchitecture, resulting in increased fragility and a heightened risk of fractures (1). Data indicate that approximately 21.7% of the domestic population meets the World Health Organization (WHO) diagnostic criteria for osteoporosis. The prevalence reaches 35.3% among postmenopausal women and 12.5% among men over 65 years of age (2). WHO has ranked osteoporosis as the second-leading cause of disability-adjusted life year (DALY) loss worldwide, following only cardiovascular diseases (3).

As a typical age-related condition, osteoporosis incidence increases exponentially with age: cross-sectional studies show a prevalence of 24.1% among individuals aged 50–64, rising to 51.8% in those over 80 (4). Patients with osteoporosis face a 3.2–6.1-fold increased risk of hip fractures (5). With the global population aging, osteoporotic fragility fractures have become a significant public health issue, with 8.9 million cases occurring worldwide each year. Among these, the 1-year mortality rate following a hip fracture is as high as 20–24%, and the disability rate exceeds 40%, imposing a considerable economic burden (6).

A critical concern is the significant gap between diagnosis and treatment: fewer than 30% of high-risk individuals are screened, and only 18.5% of patients receive standardized anti-osteoporosis therapy within 12 months following a fracture. This paradox of high disease incidence and low intervention rates severely hampers the effectiveness of osteoporosis prevention and control systems (7, 8).

Clinical management of osteoporosis adheres to a hierarchical treatment strategy, with pharmacological intervention at its core. According to the “World Health Organization Guidelines for the Prevention and Treatment of Osteoporosis,” standardized drug therapy can increase lumbar spine bone mineral density (BMD) by 6–8% and reduce the relative risk of fragility fractures by 40–70% (9). Current medications are categorized by their mechanisms of action into bone resorption inhibitors (e.g., bisphosphonates, RANKL monoclonal antibodies, estrogen modulators), bone formation promoters (e.g., parathyroid hormone analogs), dual-action agents (e.g., romosozumab), and others with alternative mechanisms (e.g., vitamin K2) (10).

Despite their efficacy in reducing vertebral fracture risk, safety concerns surrounding these medications have grown. Data from the U.S. Food and Drug Administration’s Adverse Event Reporting System (FAERS) show that 23.7% of patients discontinue treatment due to adverse drug reactions (11). This underscores the need for safe, effective, and reliable alternatives in the management of osteoporosis.

Chinese Herbal Medicine (CHM), with its multi-component and synergistic therapeutic properties, has been used clinically since the era of the Huangdi Neijing (Inner Canon of Huangdi). CHM is grounded in the traditional principle of “tonifying the kidney and strengthening the bones” to treat bone metabolism disorders. Modern clinical applications of CHM include single-herb extracts, compound formulations, and sequential combinations with bisphosphonates. However, despite numerous clinical trials investigating the anti-osteoporotic effects of CHM, many studies fall short of the Consolidated Standards of Reporting Trials (CONSORT) criteria.

While previous research has primarily focused on changes in BMD to assess CHM efficacy, data regarding its effects on bone turnover markers remain limited (12). Therefore, we conducted this systematic review and meta-analysis to comprehensively evaluate the therapeutic effectiveness of CHM in the treatment of patients with osteoporosis.

## Methods

### Data sources, search strategy, and selection criteria

This meta-analysis was conducted in full compliance with the 2020 Preferred Reporting Items for Systematic Reviews and Meta-Analyses (PRISMA) guidelines (13). Our study was registered in INPLASY platform (number: INPLASY202530115). As all data were derived from previously published studies, ethical approval and review were not required for this research.

A comprehensive literature search was performed using PubMed, Embase, Cochrane Library, China National Knowledge Infrastructure (CNKI), and Wanfang databases through March 2025. The search strategy incorporated both subject headings and free-text terms. Keywords included combinations of “Chinese herbal medicine,” “Herbal therapy,” “Traditional Chinese medicine,” “Osteoporosis,” and “Fracture” (see Supplementary File 1). Randomized controlled trials (RCTs) evaluating the effects of CHM on osteoporosis or related fractures were systematically identified. No restrictions were placed on the language of publication. Despite searching international databases, all RCTs evaluating CHM for osteoporosis were conducted in China, likely reflecting the regional focus of such research. To ensure thoroughness, reference lists of included articles were also manually reviewed to capture additional relevant studies not found during the initial database search.

A double-blind, independent screening process was employed. Two researchers independently reviewed study titles, abstracts, and full texts in a stepwise manner. Any discrepancies between reviewers were resolved by a third researcher, who served as an arbitrator to reach a consensus.

Inclusion criteria were established according to the PICOS framework:

1. Participants: Patients diagnosed with osteoporosis based on dual-energy X-ray absorptiometry (DEXA) and meeting the World Health Organization (WHO) criteria (*T*-score ≤ − 2.5) (14).
2. Interventions: The experimental group received a systematic traditional Chinese medicine regimen.
3. Comparators: The control group received standard anti-osteoporosis pharmacologic therapies or injectable treatments.
4. Outcomes: Primary outcomes included changes in BMD and bone metabolism markers.
5. Study Design: Only RCTs utilizing standardized randomization techniques (e.g., computer-generated random sequences or random number tables) were included.

### Data collection and quality assessment

Two authors independently extracted data from each study. Extracted variables included: the first author’s surname, year of publication, study location, sample size, proportion of male participants, mean patient age, clinical condition, intervention protocol, control treatment, reported outcomes, and follow-up duration. Following data extraction, both authors independently evaluated the methodological quality of each study using the Cochrane Risk of Bias Tool. Assessment domains included: random sequence generation, allocation concealment, blinding of participants and personnel, blinding of outcome assessment, completeness of outcome data, selective reporting, and other potential sources of bias (15). Any inconsistencies in data extraction or quality ratings were resolved through discussion with a third reviewer, who made final determinations based on the original study texts.

### Statistical analysis

Therapeutic outcomes of CHM were treated as continuous variables, with effect sizes expressed as weighted mean differences (WMDs) and corresponding 95% confidence intervals (CIs). Meta-analyses were conducted using a random-effects model to account for heterogeneity across studies (16, 17). Heterogeneity was assessed using the *I^2^* statistic and Cochran’s Q test, with significant heterogeneity defined as *I^2^* > 50% or a *Q*-test *p*-value < 0.10 (18, 19). Robustness of the results was evaluated via leave-one-out sensitivity analysis, in which each study was sequentially excluded to assess its impact on the overall effect size (20). Meta-regression were performed to identify potential source of heterogeneity on the basis of publication year, proportion of male participants, average patient age, clinical disease status, follow-up, and baseline BMD. Then subgroup analyses were performed and the differences between subgroups were tested using interaction t-tests, assuming normal distribution of data (21). Potential publication bias was assessed visually using funnel plot asymmetry and statistically using Egger’s linear regression test and Begg’s rank correlation test (22, 23). All analyses were conducted using STATA version 12.0 (StataCorp LLC, College Station, TX, USA), following Cochrane Collaboration guidelines for conducting and reporting meta-analyses. Two-tailed *p*-values were reported, with statistical significance set at *α* = 0.05.

## Results

### Literature search

A total of 2,240 relevant studies were initially identified through a comprehensive database search. After removing 868 duplicates using reference management software, 1,372 records remained for preliminary screening. Following title and abstract screening, 1,309 studies that did not meet the predefined eligibility criteria were excluded. The remaining 63 articles were subjected to full-text evaluation.

**Figure 1:**
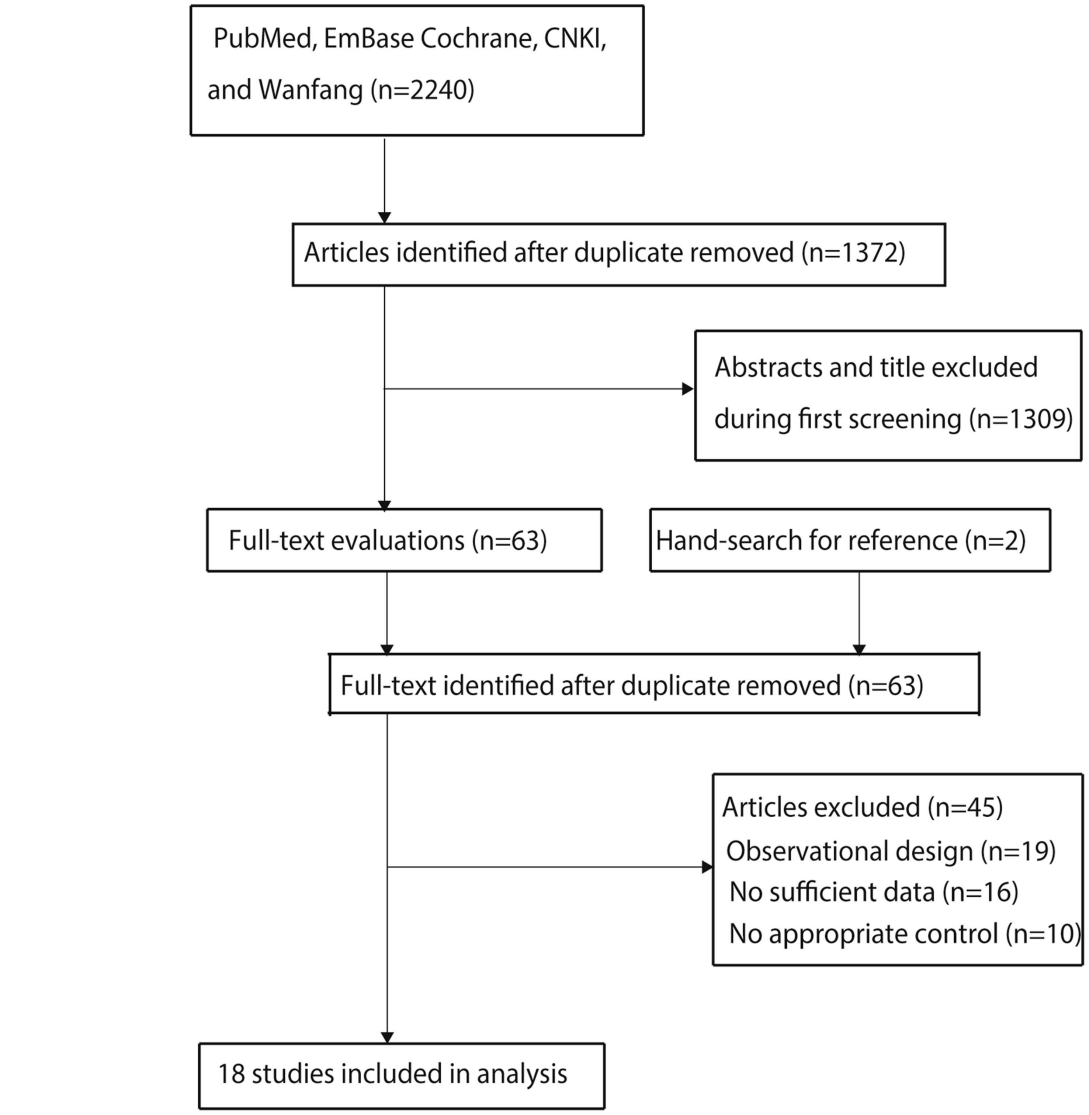
Flow diagram of the literature search and study selection process.

Through a double-blind, independent full-text review by two researchers, 18 studies were confirmed to meet the PICOS inclusion criteria and were included in the quantitative synthesis (24–41). To enhance the comprehensiveness of the literature coverage, reference lists of the included studies were manually searched. Two additional potentially eligible studies were identified. However, after duplicate verification using the CrossCheck database, both were confirmed to have already been included in the initial retrieval and were excluded due to redundancy. The final analysis included 18 studies (Figure 1).

### Study characteristics

Table 1 summarizes the basic characteristics of the included RCTs and the demographic details of the study populations. Across the 18 studies, a total of 1,816 patients diagnosed with osteoporosis were included. The median sample size of single-center studies was 98 participants (range: 62–140). Gender distribution was as follows: one study (5.6%) included only male patients, five studies (27.8%) enrolled only postmenopausal women, and 12 studies (66.6%) included mixed-gender cohorts. Regarding follow-up duration, six studies (33.3%) conducted short-term follow-up of 3 months, 11 studies (61.1%) had a 6-month follow-up, and one study (5.6%) reported outcomes at multiple time points (12 and 24 months).

**Table 1:** The baseline characteristics of identified trials and involved patients.

Study	Country	Sample size	Male (%)	Age (years)	Baseline BMD (g/cm^2^)	Disease status	Intervention	Control	Follow-up (months)
Xie 1997 (24)	China	80 (50/30)	51.3	68.1	Lumbar spine: 0.968; Femoral neck: 0.795; Greater trochanter of the femur: 0.647; Ward’s triangle area: 0.601	Primary osteoporosis with kidney-yang deficiency syndrome	Bugushengsui capsules	Vitamin D and calcium tablet	6.0
Shi 2001 (25)	China	140 (80/60)	37.1	62.3	Lumbar spine: 0.72	Primary osteoporosis with fracture	Bushenjiangu decoction	Calcium tablet	3.0
Lv 2002 (26)	China	90 (48/42)	42.2	65.1	Lumbar spine: 0.66	Primary osteoporosis	Fortified Bushenzhuangjin decoction	Active calcium cranules	3.0
Wang 2005 (27)	China	100 (50/50)	39.0	63.1	Lumbar spine: 0.67	Primary osteoporosis	Bushenzhuanggu capsules	Calcium tablet	3.0
Ke 2005 (28)	China	90 (46/44)	100.0	68.1	Lumbar spine: 0.47	Primary osteoporosis with kidney- deficiency	Bone strengthening formula	Alendronate sodium	6.0
Pan 2006 (29)	China	120 (60/60)	0.0	48.6	Lumbar spine: 0.751	Post-menopause osteoporosis	Tonifying the kidney and replenishing essence	Alendronate sodium	6.0
Luo 2008 (30)	China	215 (108/107)	37.7	65.9	Lumbar spine: 0.925; Femoral neck: 0.678; Greater trochanter of the femur: 0.668; Ward’s triangle area: 0.693	Primary osteoporosis	Strong bone granules	Calcium Gluconate	6.0
Li 2009 (31)	China	93 (47/46)	0.0	67.4	Lumbar spine: 0.290	Post-menopause osteoporosis	Yiguyin	Vitamin D and calcium tablet	6.0
Ouyang 2012 (32)	China	82 (42/40)	32.9	46.4	Lumbar spine: 0.805; Femoral neck: 0.725	Osteoporosis induced from rheumatoid arthritis	Qianggu capsules and DMARD	DMARD	6.0
Ma 2012 (33)	China	62 (31/31)	NA	NA	Lumbar spine: 0.900; Femoral neck: 0.713; Greater trochanter of the femur: 0.576; Ward’s triangle area: 0.767	Osteoporosis	Bugu capsule and Xianlinggubao	Elcatonin and alfacalcidol	12.0
Li 2013 (34)	China	80 (40/40)	0.0	59.5	NA	Post-menopause osteoporosis	Bushenhuoxue granules	Vitamin D and calcium tablet	6.0
Zhang 2013 (35)	China	80 (41/39)	0.0	66.4	Lumbar spine: 0.541	Post-menopause osteoporosis	Bushenzhuanggu decoction	Salmon Calcitonin	6.0
Liang 2014 (36)	China	100 (50/50)	47.0	62.0	Lumbar spine: 0.805	Osteoporosis	Jiangu Bushen decoction	Caltrate D tablets	3.0
Ma 2014 (37)	China	90 (45/45)	54.4	63.1	Lumbar spine: 0.52	Osteoporosis	Buzhongyiqi decoction	Salmon Calcitonin	3.0
Hu 2020 (38)	China	66 (33/33)	31.1	45.8	Lumbar spine: 0.716; Femoral neck: 0.714	Glucocorticoid-induced osteoporosis	Nourishing liver and kidney decoction	Alfacalcidol soft capsules and calcium supplement with vitamin D	6.0
Gong 2020 (39)	China	106 (53/53)	32.1	65.3	Lumbar spine: 0.39	Primary osteoporosis	Consolidating origin and improving bone formula	Calcium carbonate D3 chewable tablet and calcitriol capsules	6.0
Chen 2023 (40)	China	120 (60/60)	0.0	67.9	Lumbar spine: 0.705; Femoral neck: 0.62; Ward’s triangle area: 0.725	Post-menopause osteoporosis	Bushenzhuanggu tablet	Calcium and vitamin D	6.0
Zhang 2024 (41)	China	102 (51/51)	31.4	78.0	Lumbar spine: 0.655; Femoral neck: 0.526	Osteoporosis	Yishengushu formula	Alendronate sodium and Calcium Carbonate and Vitamin D3 Granules	3.0

Methodological quality, assessed using the Cochrane Risk of Bias Tool, indicated that the overall quality of the included studies was moderate (Table 2). The primary limitations involved allocation concealment and implementation of blinding procedures.

**Table 2:** The methodological quality assessment of included trials.

Study	Random sequence generation	Allocation concealment	Blinding of participants and personnel	Blinding of outcome assessment	Incomplete outcome data	Selective reporting	Other bias
Xie 1997 (24)	Low	Unclear	Unclear	Low	Low	High	Unclear
Shi 2001 (25)	Low	Unclear	Unclear	Low	Low	High	Unclear
Lv 2002 (26)	Low	Unclear	Unclear	Low	Low	High	Unclear
Wang 2005 (27)	Low	Unclear	Low	Low	Low	Unclear	Unclear
Ke 2005 (28)	Low	Unclear	Unclear	Low	Low	High	Unclear
Pan 2006 (29)	Low	Unclear	Unclear	Low	Low	High	Unclear
Luo 2008 (30)	Low	Unclear	Low	Low	Low	Unclear	Unclear
Li 2009 (31)	Low	Unclear	Unclear	Low	Low	High	Unclear
Ouyang 2012 (32)	Low	Unclear	Unclear	Low	Low	High	Unclear
Ma 2012 (33)	Low	Unclear	Low	Low	Low	Unclear	Unclear
Li 2013 (34)	Low	Unclear	Low	Low	Low	Unclear	Unclear
Zhang 2013 (35)	Low	Unclear	Unclear	Low	Low	High	Unclear
Liang 2014 (36)	Low	Unclear	Unclear	Low	Low	High	Unclear
Ma 2014 (37)	Low	Unclear	Low	Low	Low	Unclear	Unclear
Hu 2020 (38)	Low	Unclear	Low	Low	Low	Unclear	Unclear
Gong 2020 (39)	Low	Unclear	Low	Low	Low	Unclear	Unclear
Chen 2023 (40)	Low	Unclear	Low	Low	Low	Unclear	Unclear
Zhang 2024 (41)	Low	Unclear	Low	Low	Low	Unclear	Unclear

### BMD at various sites

**Figure 2:**
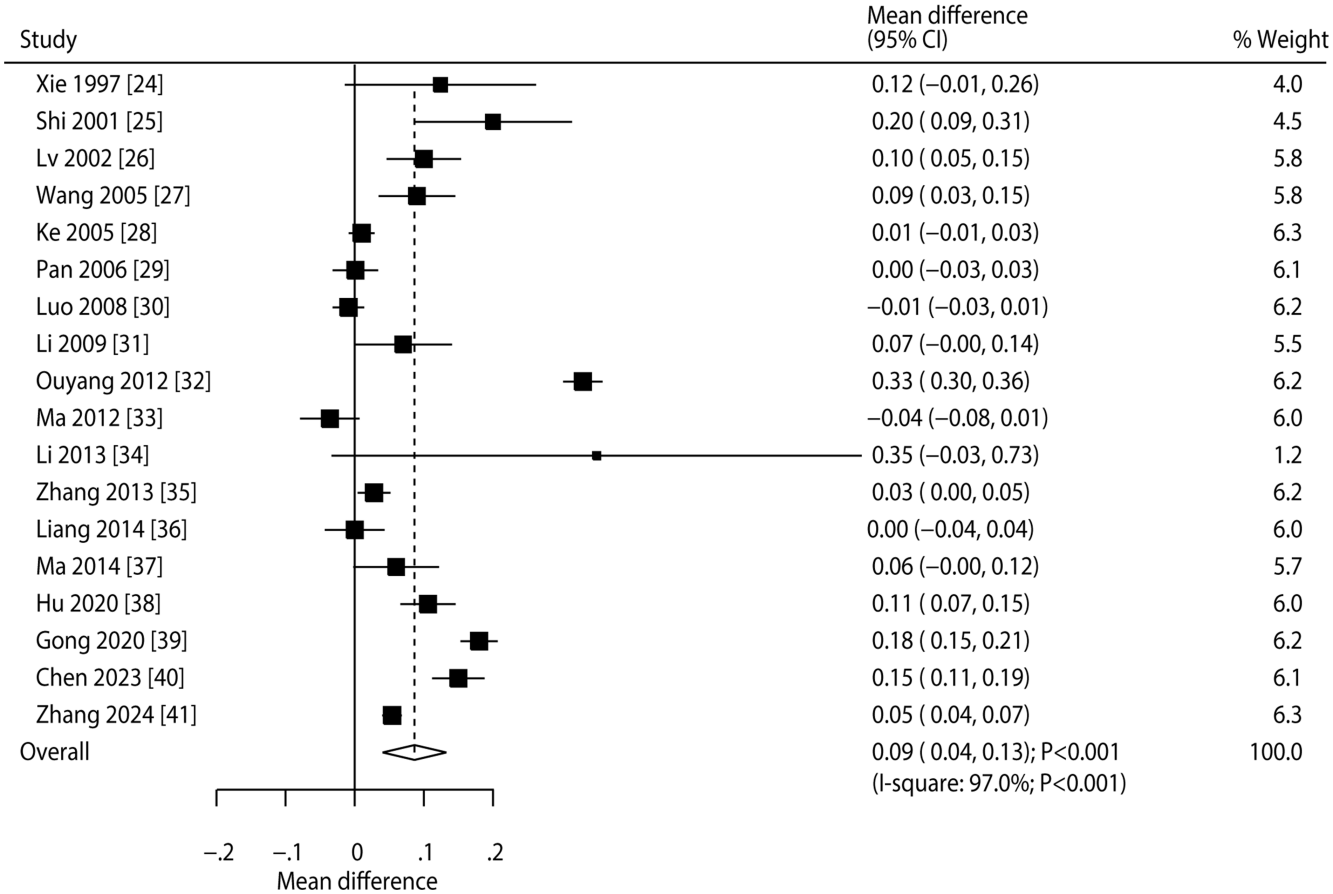
Effect of CHM on changes in BMD at the lumbar spine.

Pooled analysis of all included trials showed that CHM was associated with increased BMD at the lumbar spine compared to conventional therapy (WMD: 0.09; 95% CI: 0.04 to 0.13; *p* < 0.001; Figure 2). However, there was significant heterogeneity among studies (*I*^2^ = 97.0%; *p* < 0.001). Sensitivity analysis indicated the overall result remained stable when each study was sequentially removed (Supplementary File 2). Meta-regression found publication year, proportion of male participants, average patient age, follow-up, and baseline BMD were not significant factors contributing to the association between CHM and BMD at the lumbar spine, while clinical disease status that contributed to the association between CHM and BMD at the lumbar spine (*p* = 0.021) (Supplementary File 3). Subgroup analyses revealed that CHM significantly improved lumbar spine BMD in most subgroups. However, no statistically significant differences were observed when the proportion of male participants was ≥ 50%, the mean patient age was < 65 years, or the patients had secondary osteoporosis (Table 3). No significant publication bias was detected for lumbar spine BMD (Egger’s test *p* = 0.457; Begg’s test *p* = 0.256; Supplemental File 4).

**Table 3:** Subgroup analyses.

Outcomes	Factors	Subgroups	WMD and 95%CI	*p*-value	*I^2^* (%)	*p*-value for heterogeneity	*p*-value between subgroups
Lumbar spine	Publication year	Before 2010	0.05 (0.02 to 0.09)	0.004	80.8	<0.001	<0.001
		2010 or after	0.10 (0.03 to 0.17)	0.004	97.9	<0.001	
	Male (%)	≥ 50.0%	0.04 (−0.01 to 0.09)	0.140	57.2	0.097	<0.001
		< 50.0%	0.09 (0.04 to 0.15)	0.001	97.3	<0.001	
	Mean age (years)	≥ 65.0	0.07 (0.03 to 0.12)	0.001	95.2	<0.001	<0.001
		< 65.0	0.10 (−0.00 to 0.21)	0.052	97.7	<0.001	
	Disease status	Primary	0.06 (0.03 to 0.10)	<0.001	92.8	<0.001	<0.001
		Secondary	0.22 (−0.00 to 0.44)	0.051	98.8	<0.001	
	Follow-up (months)	3.0	0.07 (0.03 to 0.10)	<0.001	69.9	0.005	0.029
		6.0 or 12.0	0.09 (0.02 to 0.16)	0.008	98.0	<0.001	
Femoral neck	Publication year	Before 2010	0.02 (−0.05 to 0.09)	0.594	39.7	0.198	< 0.001
		2010 or after	0.11 (0.04 to 0.19)	0.003	98.7	<0.001	
	Male (%)	≥50.0%	0.09 (−0.05 to 0.23)	0.191	–	–	0.740
		<50.0%	0.09 (0.01 to 0.17)	0.020	99.3	<0.001	
	Mean age (years)	≥65.0	0.09 (0.01 to 0.17)	0.036	98.6	<0.001	<0.001
		<65.0	0.10 (−0.03 to 0.23)	0.119	98.6	<0.001	
	Disease status	Primary	0.08 (0.01 to 0.14)	0.022	97.7	<0.001	< 0.001
		Secondary	0.13 (−0.03 to 0.29)	0.116	98.9	<0.001	
	Follow-up (months)	3.0	0.08 (0.07 to 0.09)	<0.001	–	–	<0.001
		6.0 or 12.0	0.10 (0.00 to 0.19)	0.044	99.2	<0.001	
Greater trochanter of the femur	Publication year	Before 2010	0.03 (−0.02 to 0.09)	0.239	21.2	0.260	0.005
		2010 or after	−0.03 (−0.06 to 0.01)	0.118	0.0	0.818	
	Male (%)	≥50.0%	0.11 (−0.04 to 0.27)	0.155	–	–	0.231
		<50.0%	0.00 (−0.04 to 0.04)	0.961	74.8	0.019	
	Mean age (years)	≥65.0	0.03 (−0.02 to 0.09)	0.239	21.2	0.260	0.005
		<65.0	−0.03 (−0.06 to 0.01)	0.118	0.0	0.818	
	Disease status	Primary	0.01 (−0.03 to 0.05)	0.698	68.0	0.025	–
		Secondary	–	–	–	–	
	Follow-up (months)	3.0	–	–	–	–	–
		6.0 or 12.0	0.01 (−0.03 to 0.05)	0.698	68.0	0.025	
Ward’s triangle area	Publication year	Before 2010	0.07 (0.07 to 0.08)	<0.001	0.0	0.895	0.512
		2010 or after	0.09 (−0.10 to 0.27)	0.363	97.9	<0.001	
	Male (%)	≥50.0%	0.07 (−0.05 to 0.19)	0.267	–	–	0.909
		<50.0%	0.08 (0.00 to 0.16)	0.041	95.9	<0.001	
	Mean age (years)	≥65.0	0.11 (0.03 to 0.20)	0.009	91.6	<0.001	<0.001
		<65.0	−0.01 (−0.04 to 0.03)	0.636	–	–	
	Disease status	Primary	0.08 (0.01 to 0.15)	0.025	93.9	<0.001	–
		Secondary	–	–	–	–	
	Follow-up (months)	3.0	–	–	–	–	–
		6.0 or 12.0	0.08 (0.01 to 0.15)	0.025	93.9	<0.001	
ALP (u/L)	Publication year	Before 2010	0.97 (−1.33 to 3.28)	0.409	0.0	0.637	0.806
		2010 or after	0.31 (−16.79 to 17.40)	0.972	97.0	<0.001	
	Male (%)	≥50.0%	−1.72 (−10.00 to 6.56)	0.684	–	–	0.545
		<50.0%	1.30 (−7.30 to 9.90)	0.767	94.9	<0.001	
	Mean age (years)	≥65.0	−4.90 (−12.14 to 2.34)	0.184	87.9	<0.001	<0.001
		< 65.0	9.55 (−2.70 to 21.80)	0.127	93.0	<0.001	
	Disease status	Primary	−3.81 (−9.93 to 2.32)	0.223	86.8	<0.001	<0.001
		Secondary	16.17 (1.02 to 31.32)	0.036	93.3	<0.001	
	Follow-up (months)	3.0	–	–	–	–	–
		6.0 or 12.0	0.98 (−6.88 to 8.83)	0.808	94.2	<0.001	
Serum Ca	Publication year	Before 2010	−0.00 (−0.03 to 0.03)	0.945	33.6	0.211	<0.001
		2010 or after	0.13 (−0.23 to 0.50)	0.481	99.4	<0.001	
	Male (%)	≥50.0%	0.10 (−0.05 to 0.25)	0.186	–	–	0.817
		<50.0%	0.08 (−0.11 to 0.26)	0.426	99.0	<0.001	
	Mean age (years)	≥65.0	−0.05 (−0.17 to 0.06)	0.359	84.7	<0.001	<0.001
		< 65.0	0.21 (−0.07 to 0.50)	0.143	99.4	<0.001	
	Disease status	Primary	−0.02 (−0.08 to 0.03)	0.444	78.6	<0.001	<0.001
		Secondary	0.41 (−0.22 to 1.05)	0.202	99.7	<0.001	
	Follow-up (months)	3.0	0.01 (−0.04 to 0.06)	0.711	–	–	0.004
		6.0 or 12.0	0.09 (−0.11 to 0.29)	0.386	99.0	<0.001	
Serum P	Publication year	Before 2010	0.02 (−0.06 to 0.11)	0.572	0.0	0.446	0.005
		2010 or after	−0.09 (−0.31 to 0.13)	0.405	98.2	<0.001	
	Male (%)	≥50.0%	–	–	–	–	–
		< 50.0%	−0.05 (−0.22 to 0.12)	0.574	97.2	< 0.001	
	Mean age (years)	≥65.0	−0.11 (−0.47 to 0.24)	0.531	97.4	<0.001	<0.001
		<65.0	0.01 (−0.04 to 0.06)	0.665	55.9	0.104	
	Disease status	Primary	−0.09 (−0.35 to 0.16)	0.475	97.8	<0.001	<0.001
		Secondary	0.04 (−0.04 to 0.11)	0.376	59.5	0.116	
	Follow-up (months)	3.0	−0.02 (−0.07 to 0.03)	0.385	–	–	<0.001
		6.0 or 12.0	−0.05 (−0.28 to 0.17)	0.648	97.5	<0.001	

**Figure 3:**
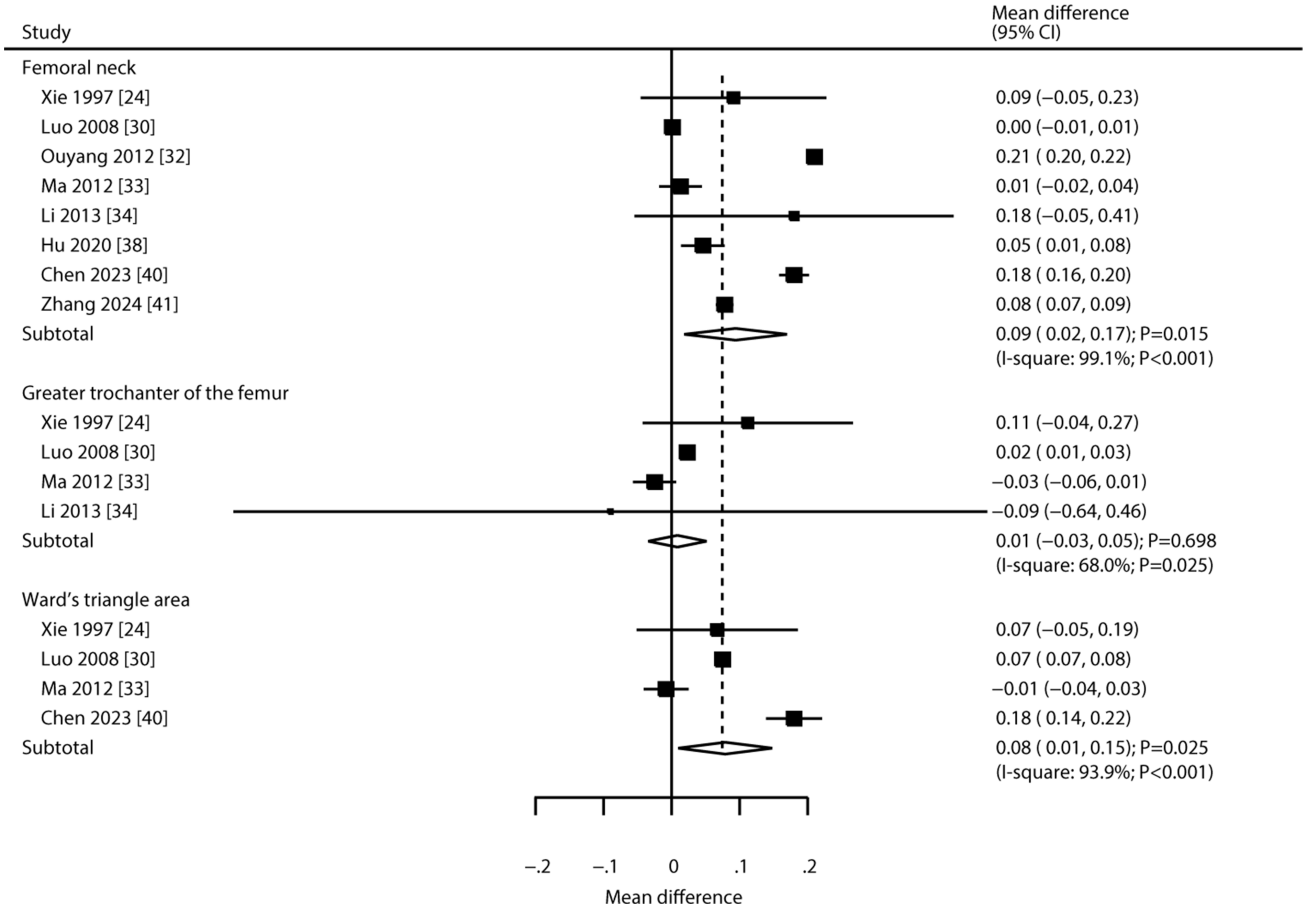
Effect of CHM on changes in BMD at the femoral neck, greater trochanter of the femur, and Ward’s triangle area.

Eight, four, and four trials, respectively, reported on CHM’s effects on BMD at the femoral neck, greater trochanter of the femur, and Ward’s triangle area (Figure 3). Pooled results indicated CHM was associated with higher BMD at the femoral neck (WMD: 0.09; 95% CI: 0.02 to 0.17; *p* = 0.015) and Ward’s triangle area (WMD: 0.08; 95% CI: 0.01 to 0.15; *p* = 0.025). However, CHM was not associated with changes in BMD at the greater trochanter of the femur (WMD: 0.01; 95% CI: −0.03 to 0.05; *p* = 0.698). Significant heterogeneity was noted for BMD at the femoral neck (*I^2^* = 99.1%; *p* < 0.001), greater trochanter (*I^2^* = 68.0%; *p* = 0.025), and Ward’s triangle area (*I^2^* = 93.9%; *p* < 0.001). Sensitivity analyses suggested variable stability of results, likely due to the small number of included trials and wide confidence intervals (Supplementary File 2). Meta-regression analyses found publication year, proportion of male participants, average patient age, clinical disease status, follow-up, and baseline BMD were not significant factors contributing to the association between CHM and BMD at the femoral neck, greater trochanter of the femur, and Ward’s triangle area (Supplementary File 3). Subgroup analyses found that CHM significantly increased femoral neck BMD in studies published in 2010 or later, with <50% male participants, mean age ≥ 65 years, patients with primary osteoporosis, and across all follow-up durations. CHM did not affect BMD at the greater trochanter in any subgroup. For Ward’s triangle area, significant improvements were seen in studies published before 2010, with <50% male participants, mean age ≥ 65 years, patients with primary osteoporosis, and follow-up durations of 6 or 12 months (Table 3). No significant publication bias was observed for BMD at the femoral neck (Egger’s test *p* = 0.658; Begg’s test *p* = 0.711), greater trochanter (Egger’s *p* = 0.787; Begg’s *p* = 0.734), or Ward’s triangle area (Egger’s *p* = 0.965; Begg’s *p* = 1.000; Supplemental File 4).

### Bone turnover markers

**Figure 4:**
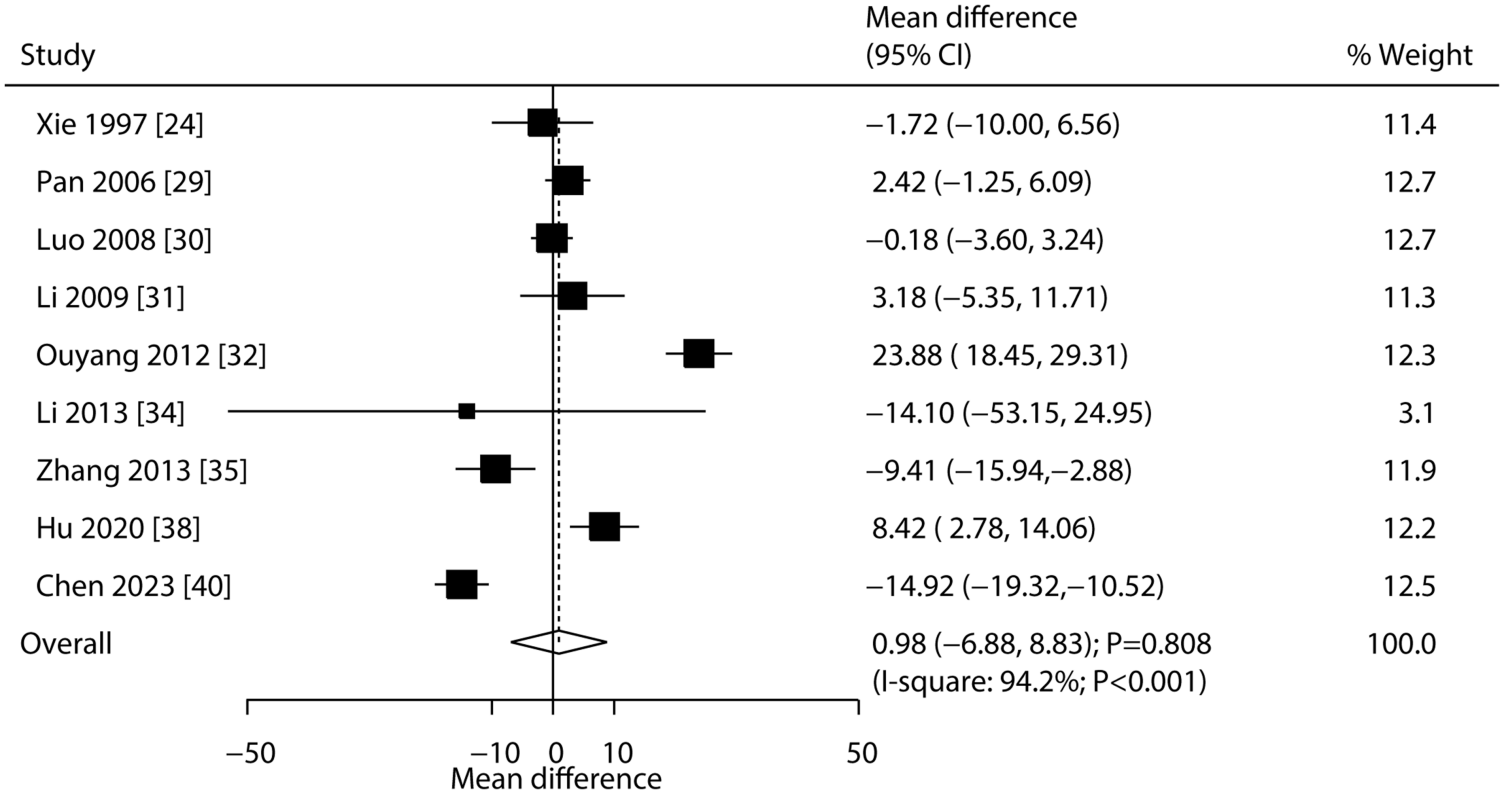
Effect of CHM on changes in alkaline phosphatase levels.

Nine studies reported the effect of CHM on alkaline phosphatase. The pooled result showed no significant association between CHM and alkaline phosphatase levels (WMD: 0.98; 95% CI: −6.88 to 8.83; *p* = 0.808; Figure 4). Notably, there was significant heterogeneity across studies (*I*^2^ = 94.2%; *p* < 0.001). Sensitivity analysis confirmed the overall conclusion was stable, with no individual study altering the pooled effect (Supplementary File 2). Meta-regression analyses found average patient age (*p* = 0.029) and disease status (*p* = 0.018) contributed to the association between CHM and alkaline phosphatase levels (Supplementary File 3). Subgroup analysis indicated CHM was associated with increased alkaline phosphatase levels in patients with secondary osteoporosis (Table 3). No significant publication bias was detected (Egger’s test *p* = 0.933; Begg’s test *p* = 0.917; Supplementary File 4).

**Figure 5:**
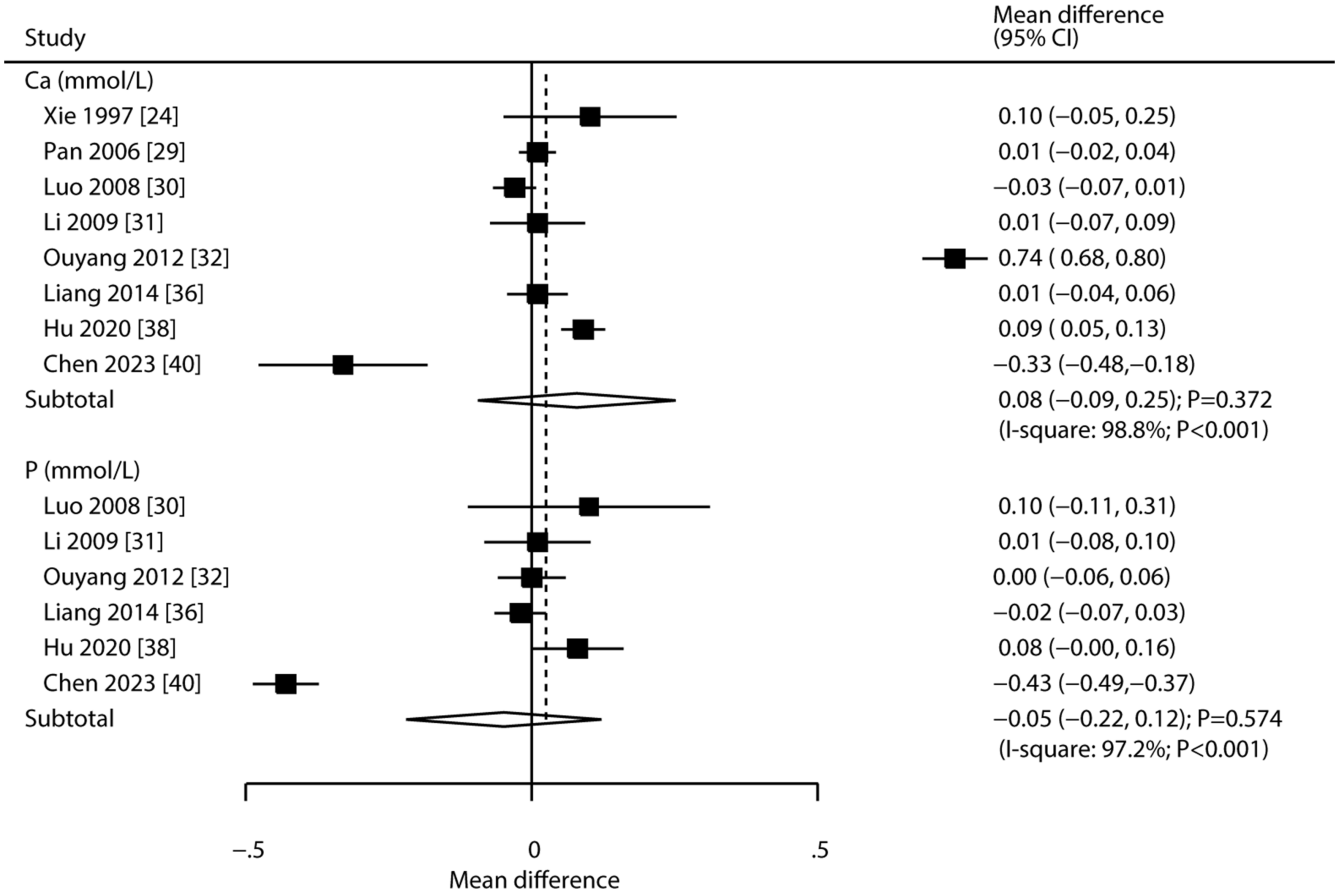
Effect of CHM on changes in serum calcium and serum phosphorus levels.

Eight and six studies reported the effects of CHM on serum calcium and serum phosphorus, respectively (Figure 5). CHM had no significant effect on serum calcium (WMD: 0.08; 95% CI: −0.09 to 0.25; *p* = 0.372) or serum phosphorus (WMD: -0.05; 95% CI: −0.22 to 0.12; *p* = 0.574). Substantial heterogeneity was observed for both serum calcium (*I^2^* = 98.8%; *p* < 0.001) and phosphorus (*I^2^* = 97.2%; *p* < 0.001). Sensitivity analysis indicated stable pooled conclusions for serum calcium, while the effect on serum phosphorus was more variable (Supplementary File 2). Meta-regression analyses found publication year, proportion of male participants, average patient age, clinical disease status, follow-up, and baseline BMD were not significant factors contributing to the association of CHM with serum calcium and serum phosphorus (Supplementary File 3). Subgroup analyses showed CHM was not associated with serum calcium or phosphorus levels in any subgroup (Table 3). No significant publication bias was found for either outcome (serum calcium: Egger’s *p* = 0.832, Begg’s *p* = 0.536; serum phosphorus: Egger’s *p* = 0.694, Begg’s *p* = 0.707; Supplementary File 4).

## Discussion

This systematic review included 18 RCTs, encompassing 1,816 patients with osteoporosis, identified through rigorous screening and quantitative synthesis. The meta-analysis demonstrated that CHM significantly improved BMD at the lumbar spine, femoral neck, and Ward’s triangle area when compared with conventional therapies. However, no statistically significant differences were observed in BMD at the greater trochanter. Secondary outcomes indicated no clinically meaningful differences in bone turnover markers, including alkaline phosphatase, serum calcium, and serum phosphorus levels. The methodological limitations of included studies, specifically unclear allocation concealment and inadequate blinding, introduce potential performance and detection biases. In trials comparing CHM to conventional therapy, lack of blinding may lead to observer bias in assessing BMD changes, particularly if evaluators were aware of treatment assignments. Additionally, unblinded participants might report outcomes subjectively, potentially overestimating CHM efficacy.

Regarding BMD outcomes, CHM was found to significantly increase lumbar spine BMD in most cases. Additionally, CHM also improved BMD at the femoral neck and Ward’s triangle area, but had no significant effect at the greater trochanter. CHM’s osteoprotective effects are attributed to bioactive components such as flavonoids, saponins, and polysaccharides, supported by preclinical studies: (1) Flavonoids: *In vitro* studies show icariin activates the Wnt/*β*-catenin pathway in osteoblasts, increasing Runx2 expression and promoting bone formation (42). Animal models of osteoporosis demonstrate icariin reduces bone loss via suppression of NF-κB/RANKL signaling in osteoclasts (43); (2) Saponins: These compounds enhance osteoblast differentiation by upregulating BMP-2/Smad signaling, as shown in murine pre-osteoblastic cells (44). Moreover, osteoporosis exhibited improved BMD after notoginsenoside R1 treatment, associated with decreased TNF-*α* levels (45); and (3) Polysaccharides: *In vitro*, these promote mesenchymal stem cell osteogenesis through Wnt/β-catenin pathway activation, while inhibiting adipogenesis (46). A rat model of postmenopausal osteoporosis showed Astragalus polysaccharides increased trabecular bone density via estrogen receptor α-mediated signaling (47). For secondary osteoporosis, CHM’s immunomodulatory effects on alkaline phosphatase may involve: (1) Triptolide: This component suppresses NF-κB signaling in activated T cells, reducing TNF-α-induced osteoclastogenesis (48); (2) Curcuminoids: These enhance BMP-2 expression in osteoblasts while inhibiting RANKL secretion from immune cells (49); and (3) Glycyrrhizic acid: This compound modulates the Th17/Treg balance, reducing IL-17-mediated bone resorption (50).

This review also found that patient heterogeneity may influence the therapeutic efficacy of CHM, particularly in the following contexts: (1) Male patients with osteoporosis—Bone loss in men is largely attributed to age-related testosterone decline. Phytoestrogen components in CHM may have limited effects on androgen-regulated bone metabolism (51). Furthermore, men tend to have a higher proportion of cortical bone and lower bone turnover rates, which may attenuate the effects of CHM, especially when its benefits are more pronounced in trabecular bone (52). (2) Younger patients—In this population, active bone remodeling may make them more responsive to conventional anti-resorptive therapies, whereas the gradual regulatory effects of CHM may require longer durations to manifest (53). (3) Patients with secondary osteoporosis—Secondary osteoporosis, often linked to glucocorticoid use or endocrine disorders, involves complex mechanisms such as inflammatory cytokine overactivation. Standard CHM formulations may be insufficient to specifically target these inflammatory pathways (54).

The pooled analysis showed no significant effects of CHM on key bone turnover markers, including alkaline phosphatase, serum calcium, and serum phosphorus. These findings suggest that CHM, in its conventional forms, may not markedly influence the systemic balance between osteoblast-driven bone formation and osteoclast-mediated resorption. While alkaline phosphatase serves as a surrogate for osteoblast activity, and calcium/phosphorus levels reflect mineralization processes, the pleiotropic effects of CHM—primarily involving Wnt/*β*-catenin activation and RANKL/OPG axis modulation—may not translate into measurable changes in these biomarkers at a population level (55, 56).

However, subgroup analysis revealed that patients with secondary osteoporosis receiving CHM showed elevated alkaline phosphatase levels. This may reflect CHM’s immunomodulatory potential in inflammatory environments, as seen in secondary osteoporosis. Bioactive components such as triptolide and curcuminoids may suppress NF-κB signaling, reduce TNF-*α*-driven osteoclastogenesis, and promote BMP/Smad-mediated osteoblast differentiation (57, 58).

This study has several methodological limitations that should be considered when interpreting the results: (1) notably, all included studies were conducted in China, introducing significant geographical and potential ethnic biases. This limitation may affect the generalizability of our findings to populations with different genetic backgrounds, lifestyle factors, and healthcare practices. Future research should prioritize multinational, multilingual RCTs to evaluate CHM efficacy in diverse populations. Standardizing CHM formulations and reporting herb compositions in detail will facilitate cross-cultural comparisons and enhance evidence generalizability; (2) Follow-up durations in included studies ranged from 3 to 12 months, with only 1 study lasting 12 months. This short-term observation may underestimate CHM’s long-term effects on bone remodeling and fracture risk. Osteoporosis treatment typically requires ≥2 years to demonstrate significant fracture risk reduction. Additionally, rare adverse events may not emerge within short follow-ups, necessitating long-term safety surveillance; (3) despite conducting sensitivity and subgroup analyses, residual heterogeneity remained; (4) the marginal statistical significance observed in BMD improvements at the greater trochanter and in bone marker changes reflects limited statistical power; post-hoc analysis suggested inadequate power to detect small but potentially relevant differences; and (5) as with all meta-analyses based on published literature, there is a risk of publication bias and limitations in the depth of individual patient data, which restricted the ability to perform more granular analyses.

## Conclusion

Our findings suggest that CHM may offer adjunctive benefit in improving BMD at specific skeletal sites among osteoporosis patients. However, the moderate-quality evidence, high heterogeneity, and regional limitations of included studies necessitate cautious interpretation. CHM’s efficacy relative to conventional therapies remains uncertain and requires validation by large, multicenter RCTs with rigorous methodology.

## Data Availability

The original contributions presented in the study are included in the article/Supplementary material, further inquiries can be directed to the corresponding author/s.
