# Supplementary material for: Efficacy of Chinese herbal medicine in patients with osteoporosis: a systematic review and meta-analysis
Source: Front Med (Lausanne). 2025 Jul 25;12:1620264. doi: 10.3389/fmed.2025.1620264 (PMC12331595; doi:10.3389/fmed.2025.1620264)
Supplement: Supplementary file 1 [file Table_1.doc]

Search strategy in PubMed:

("Chinese herbal medicine"[TIAB] OR "Herbal therapy"[TIAB] OR "Traditional Chinese medicine"[TIAB] OR "CHM"[TIAB] OR "TCM"[TIAB]) AND ("Osteoporosis"[MeSH] OR "Bone loss"[TIAB] OR "Bone density"[MeSH] OR "DXA"[TIAB]) AND ("Randomized controlled trial"[PT] OR "Clinical trial"[PT])
