# Supplementary material for: Efficacy of Chinese herbal medicine in patients with osteoporosis: a systematic review and meta-analysis
Source: Front Med (Lausanne). 2025 Jul 25;12:1620264. doi: 10.3389/fmed.2025.1620264 (PMC12331595; doi:10.3389/fmed.2025.1620264)
Supplement: Supplementary file 2 [file Table_2.DOC]

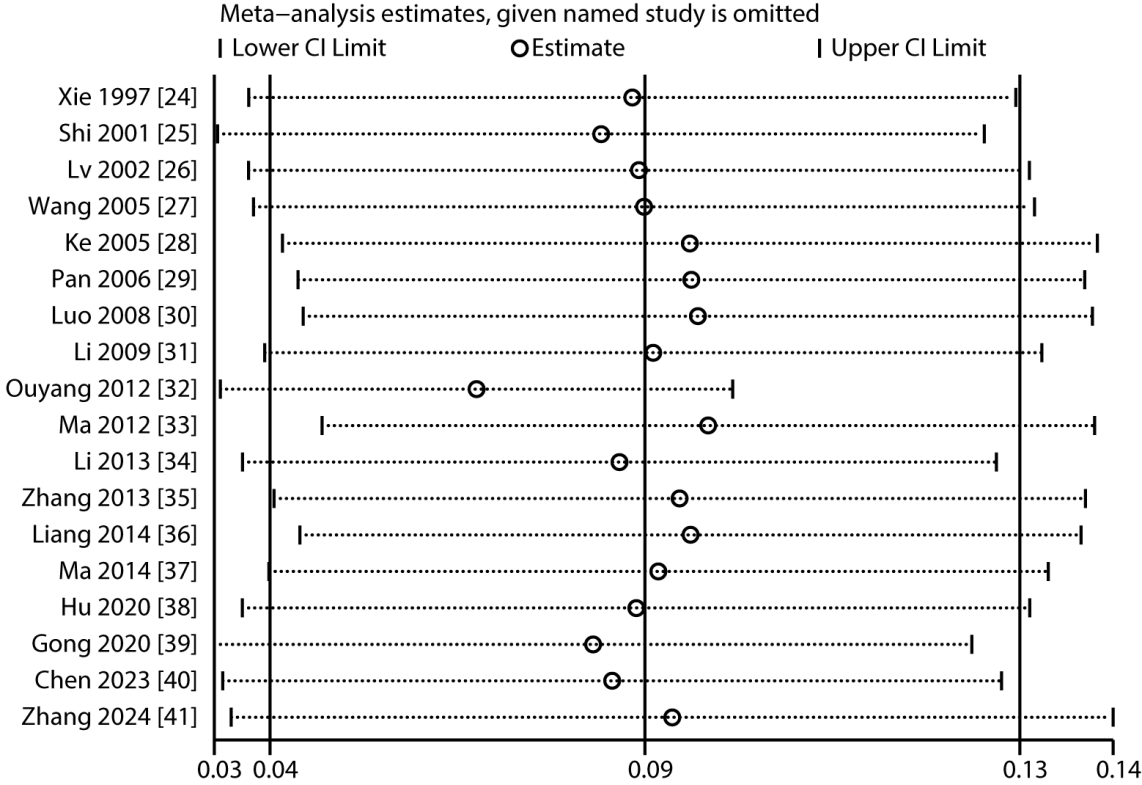


Figure S1. Sensitivity analysis for BMD at lumbar spine


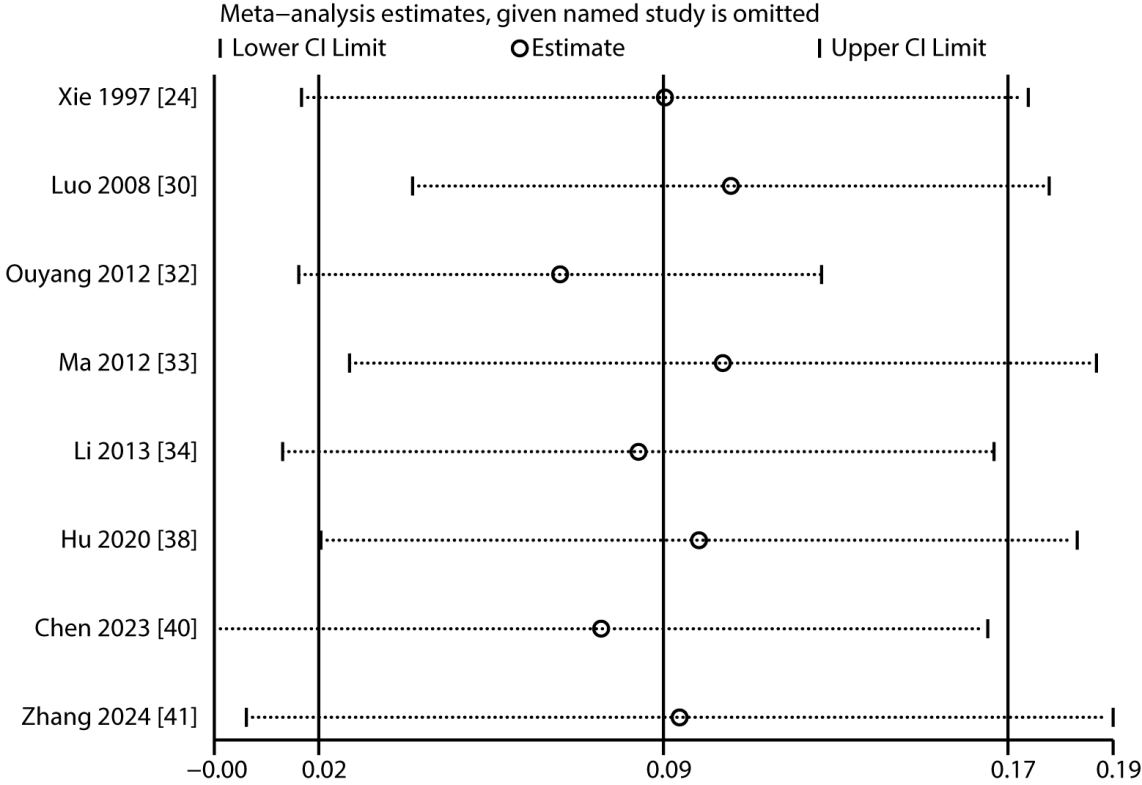


Figure S2. Sensitivity analysis for BMD at femoral neck


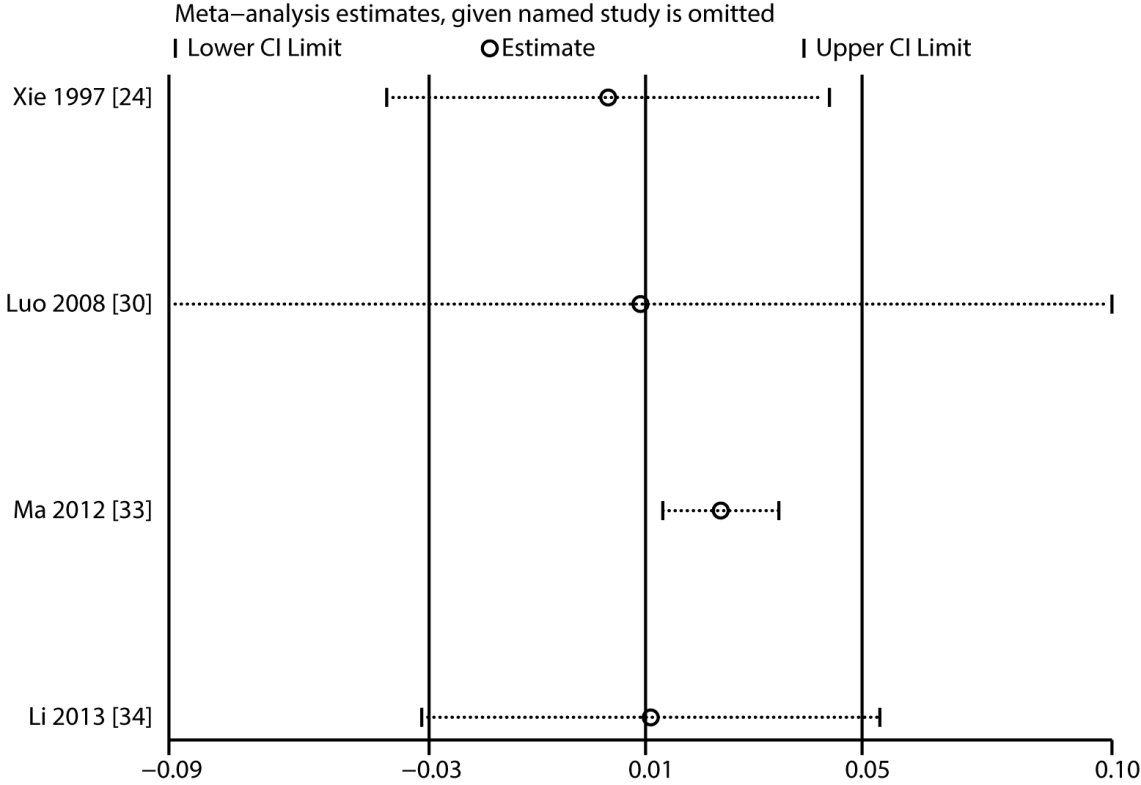


Figure S3. Sensitivity analysis for BMD at Greater trochanter of the femur


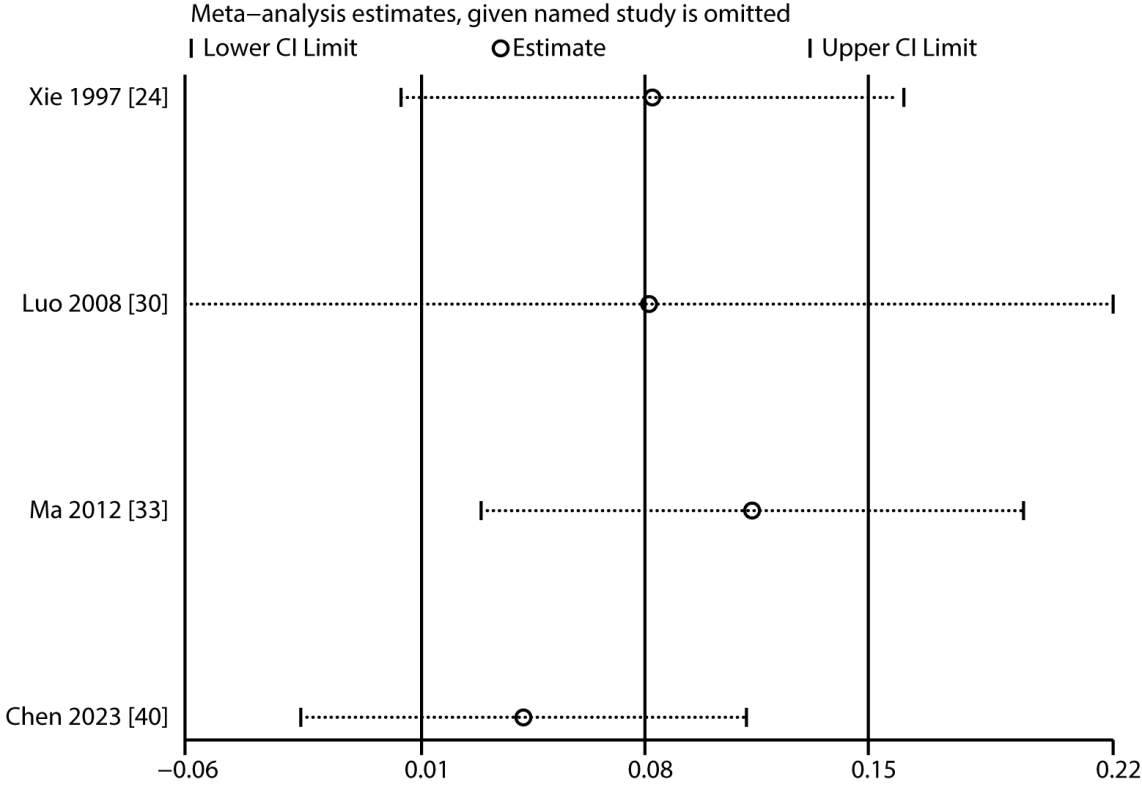


Figure S4. Sensitivity analysis for BMD at Ward’s triangle area


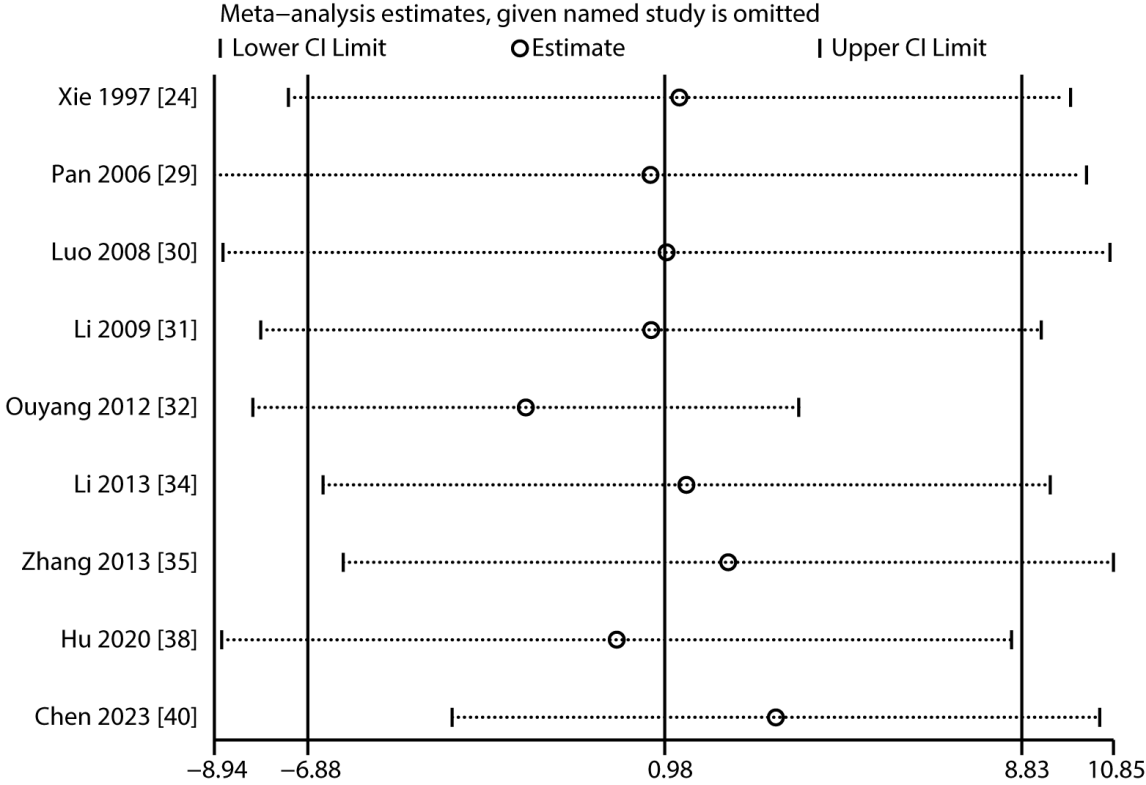


Figure S5. Sensitivity analysis for ALP


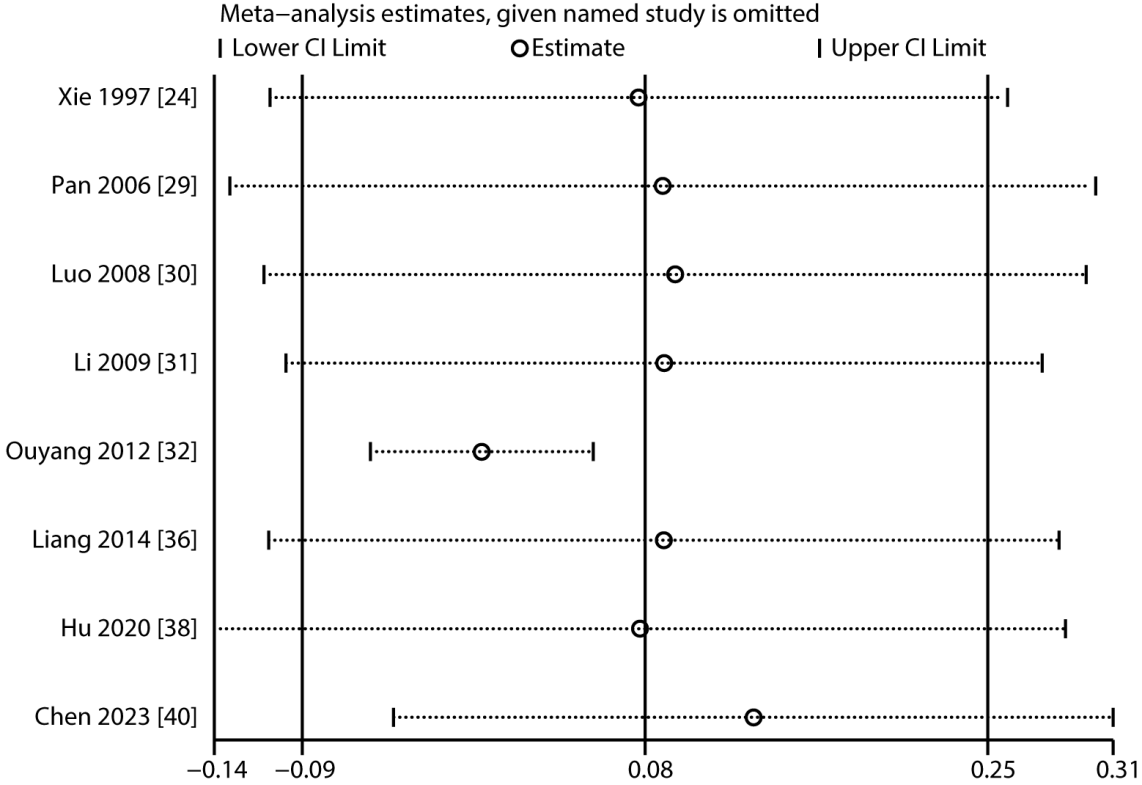


Figure S6. Sensitivity analysis for serum calcium


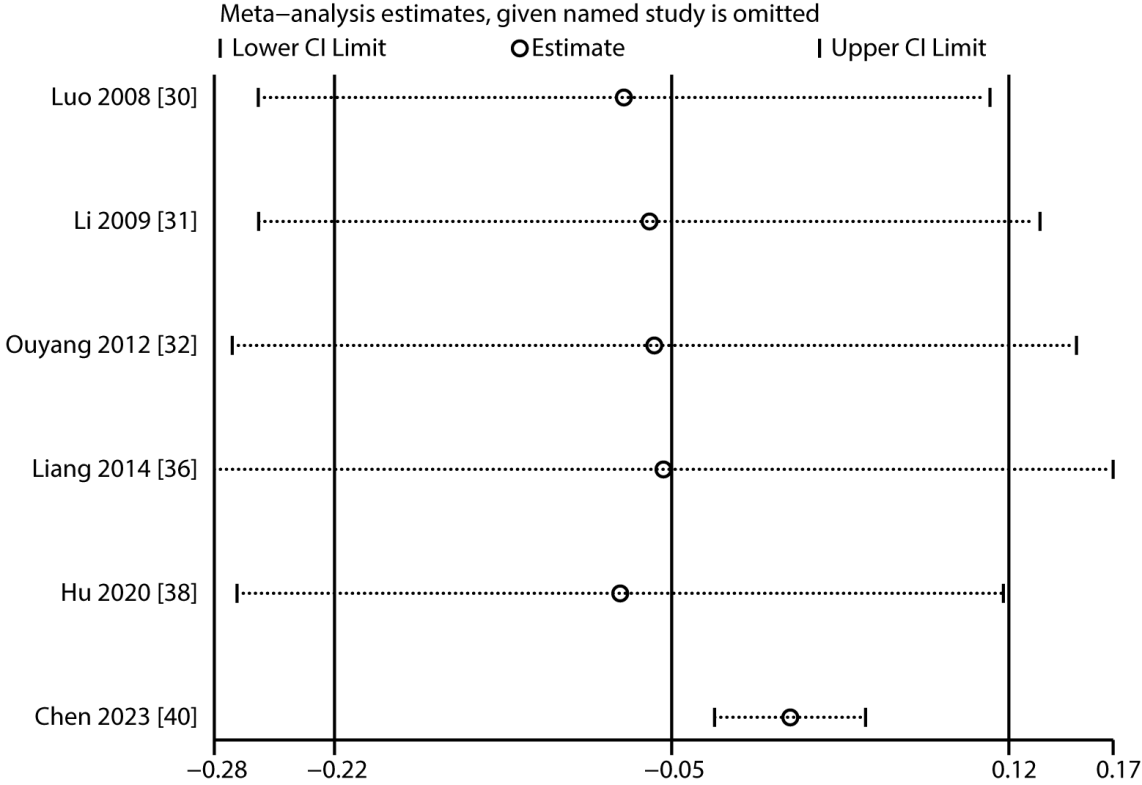


Figure S7. Sensitivity analysis for serum P
