# Supplementary material for: Efficacy of Chinese herbal medicine in patients with osteoporosis: a systematic review and meta-analysis
Source: Front Med (Lausanne). 2025 Jul 25;12:1620264. doi: 10.3389/fmed.2025.1620264 (PMC12331595; doi:10.3389/fmed.2025.1620264)
Supplement: Supplementary file 3 [file Table_3.DOCX]

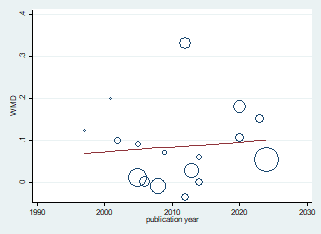


Figure S1. Meta-regression of publication year on BMD at the lumbar spine (P=0.726)


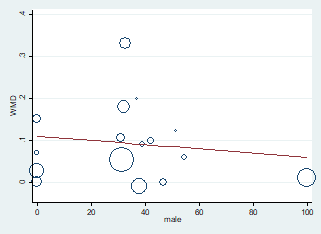


Figure S2. Meta-regression of proportion of male participants on BMD at the lumbar spine (P=0.603)


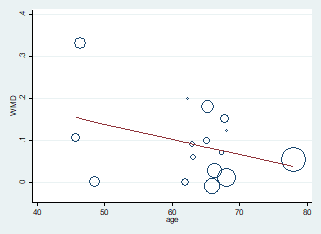


Figure S3. Meta-regression of average patient age on BMD at the lumbar spine (P=0.193)


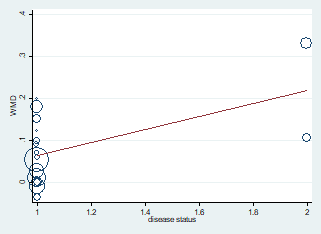


Figure S4. Meta-regression of disease status on BMD at the lumbar spine (P=0.021)


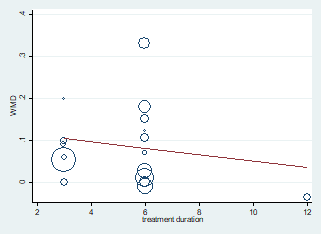


Figure S5. Meta-regression of follow-up on BMD at the lumbar spine (P=0.489)


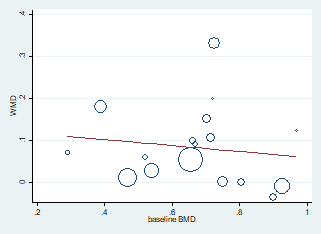


Figure S6. Meta-regression of baseline BMD on BMD at the lumbar spine (P=0.595)


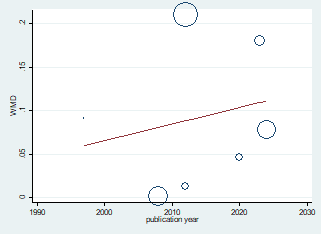


Figure S7. Meta-regression of publication year on BMD at the femoral neck (P=0.660)


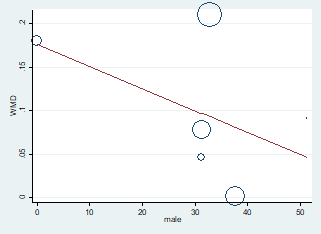


Figure S8. Meta-regression of proportion of male participants on BMD at the femoral neck (P=0.265)


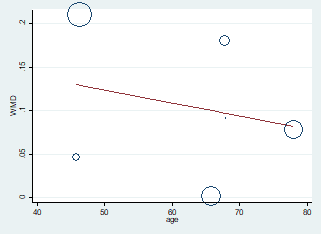


Figure S9. Meta-regression of average patient age on BMD at the femoral neck (P=0.644)


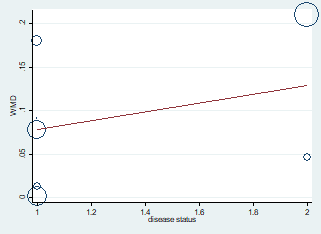


Figure S10. Meta-regression of disease status on BMD at the femoral neck (P=0.494)


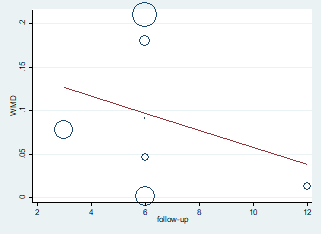


Figure S11. Meta-regression of follow-up on BMD at the femoral neck (P=0.472)


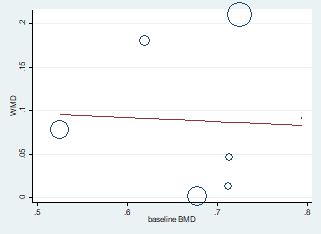


Figure S12. Meta-regression of baseline BMD on BMD at the femoral neck (P=0.923)


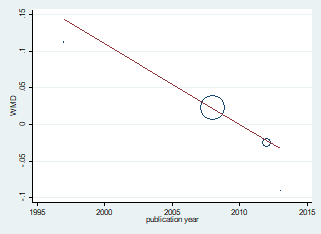


Figure S13. Meta-regression of publication year on BMD at the greater trochanter of the femur (P=0.094)


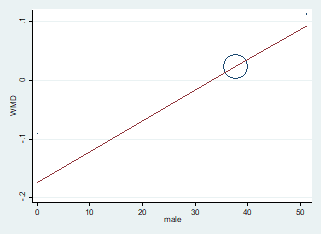


Figure S14. Meta-regression of proportion of male participants on BMD at the greater trochanter of the femur (P=0.460)


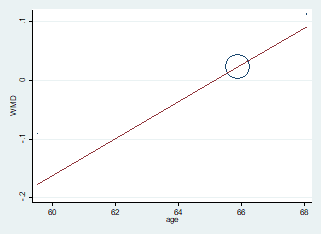


Figure S15. Meta-regression of average patient age on BMD at the greater trochanter of the femur (P=0.462)


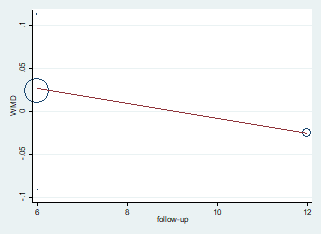


Figure S16. Meta-regression of follow-up on BMD at the greater trochanter of the femur (P=0.198)


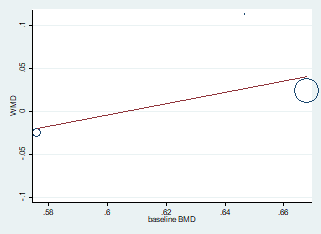


Figure S17. Meta-regression of baseline BMD on BMD at the greater trochanter of the femur (P=0.542)


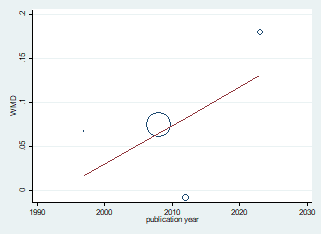


Figure S18. Meta-regression of publication year on BMD at the Ward’s triangle area (P=0.450)


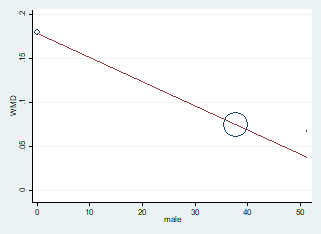


Figure S19. Meta-regression of proportion of male participants on BMD at the Ward’s triangle area (P=0.129)


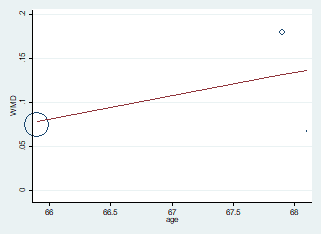


Figure S20. Meta-regression of average patient age on BMD at the Ward’s triangle area (P=0.656)


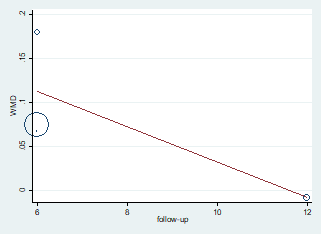


Figure S21. Meta-regression of follow-up on BMD at the Ward’s triangle area (P=0.243)


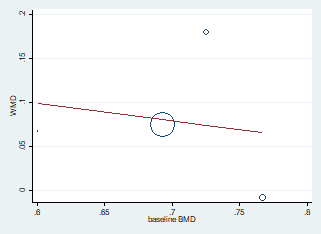


Figure S22. Meta-regression of baseline BMD on BMD at the Ward’s triangle area (P=0.832)


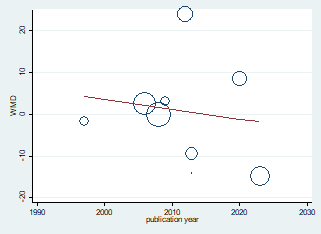


Figure S23. Meta-regression of publication year on ALP (P=0.700)


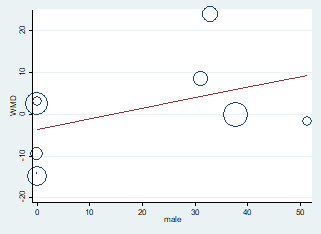


Figure S24. Meta-regression of proportion of male participants on ALP (P=0.241)


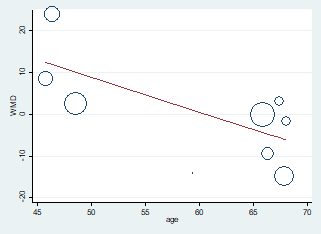


Figure S25. Meta-regression of average patient age on ALP (P=0.029)


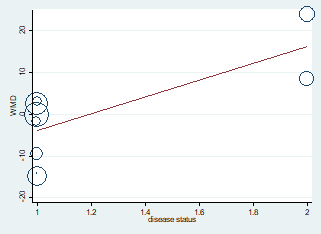


Figure S26. Meta-regression of disease status on ALP (P=0.018)


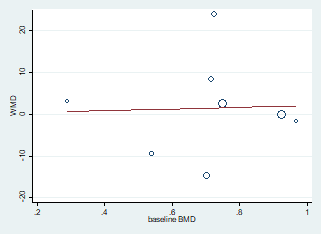


Figure S27. Meta-regression of baseline BMD on ALP (P=0.946)


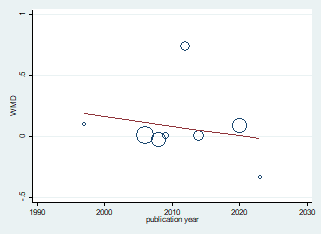


Figure S28. Meta-regression of publication year on Ca (P=0.609)


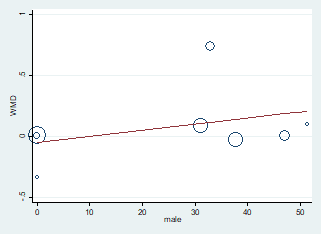


Figure S29. Meta-regression of proportion of male participants on Ca (P=0.384)


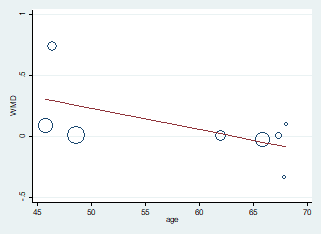


Figure S30. Meta-regression of average patient age on Ca (P=0.123)


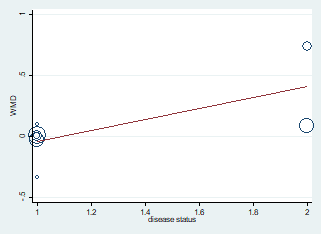


Figure S31. Meta-regression of disease status on Ca (P=0.053)


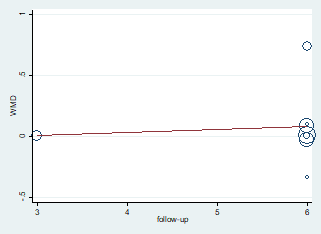


Figure S32. Meta-regression of follow-up on Ca (P=0.830)


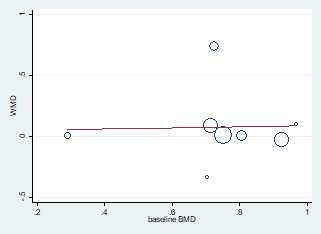


Figure S33. Meta-regression of baseline BMD on Ca (P=0.940)


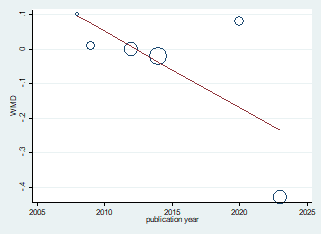


Figure S34. Meta-regression of publication year on P (P=0.156)


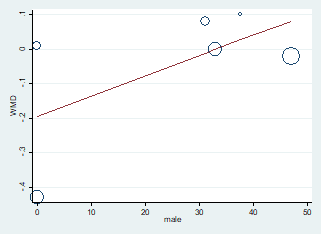


Figure S35. Meta-regression of proportion of male participants on P (P=0.216)


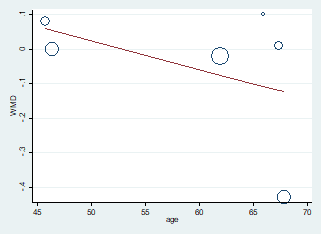


Figure S36. Meta-regression of average patient age on P (P=0.385)


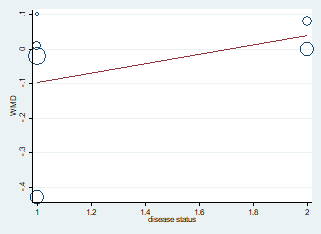


Figure S37. Meta-regression of disease status on P (P=0.487)


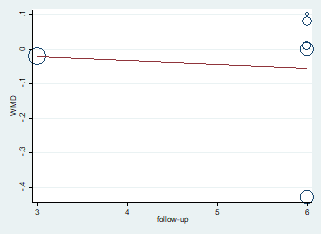


Figure S38. Meta-regression of follow-up on P (P=0.892)


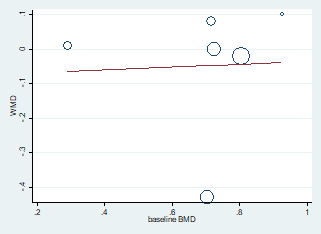


Figure S39. Meta-regression of baseline BMD on P (P=0.939)
