# Supplementary material for: Efficacy of Chinese herbal medicine in patients with osteoporosis: a systematic review and meta-analysis
Source: Front Med (Lausanne). 2025 Jul 25;12:1620264. doi: 10.3389/fmed.2025.1620264 (PMC12331595; doi:10.3389/fmed.2025.1620264)
Supplement: Supplementary file 4 [file Table_4.DOC]

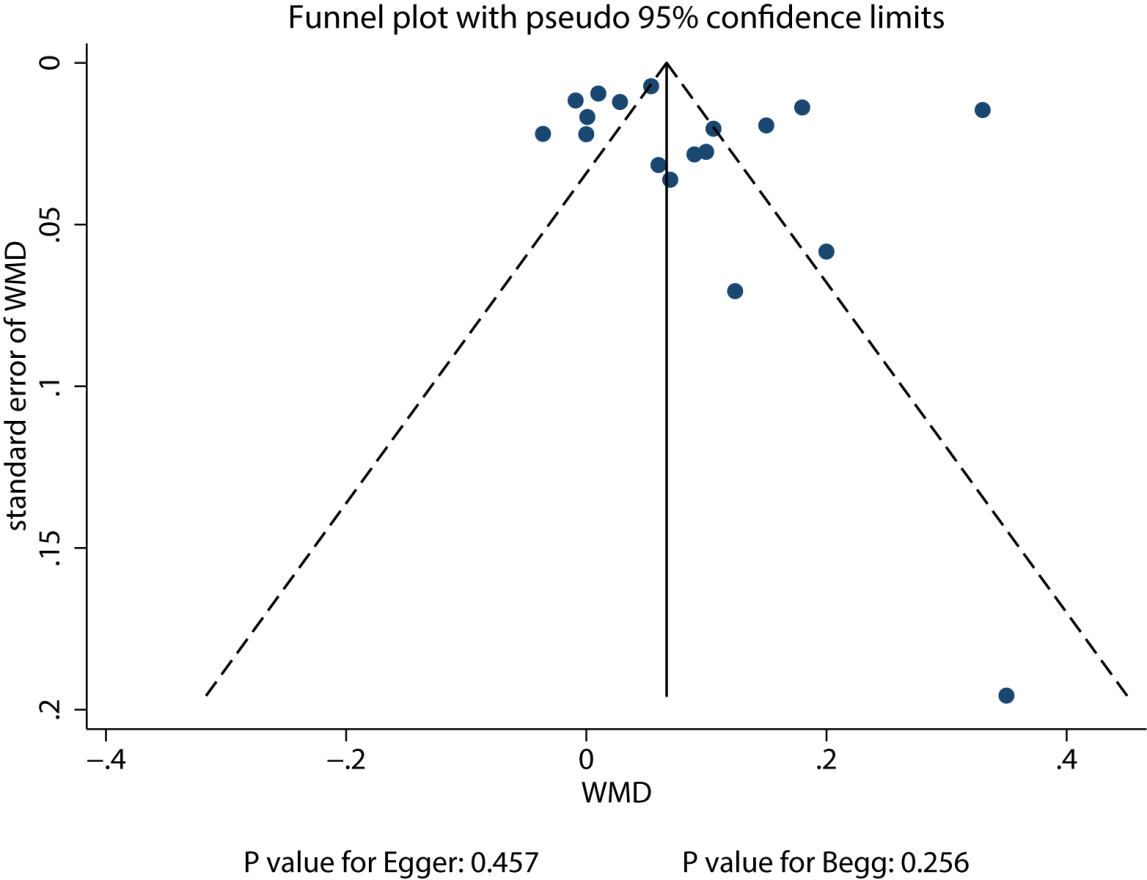


Figure S1. Funnel plot for BMD at lumbar spine


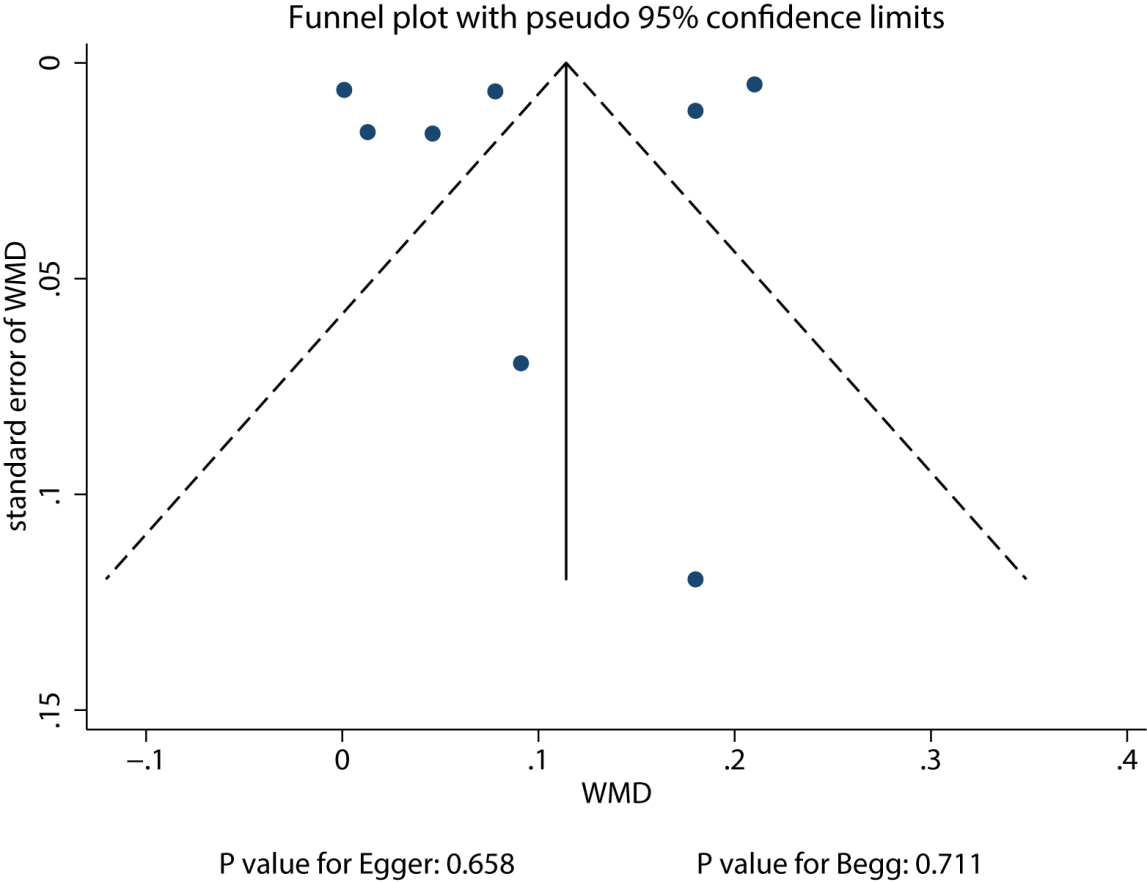


Figure S2. Funnel plot for BMD at femoral neck


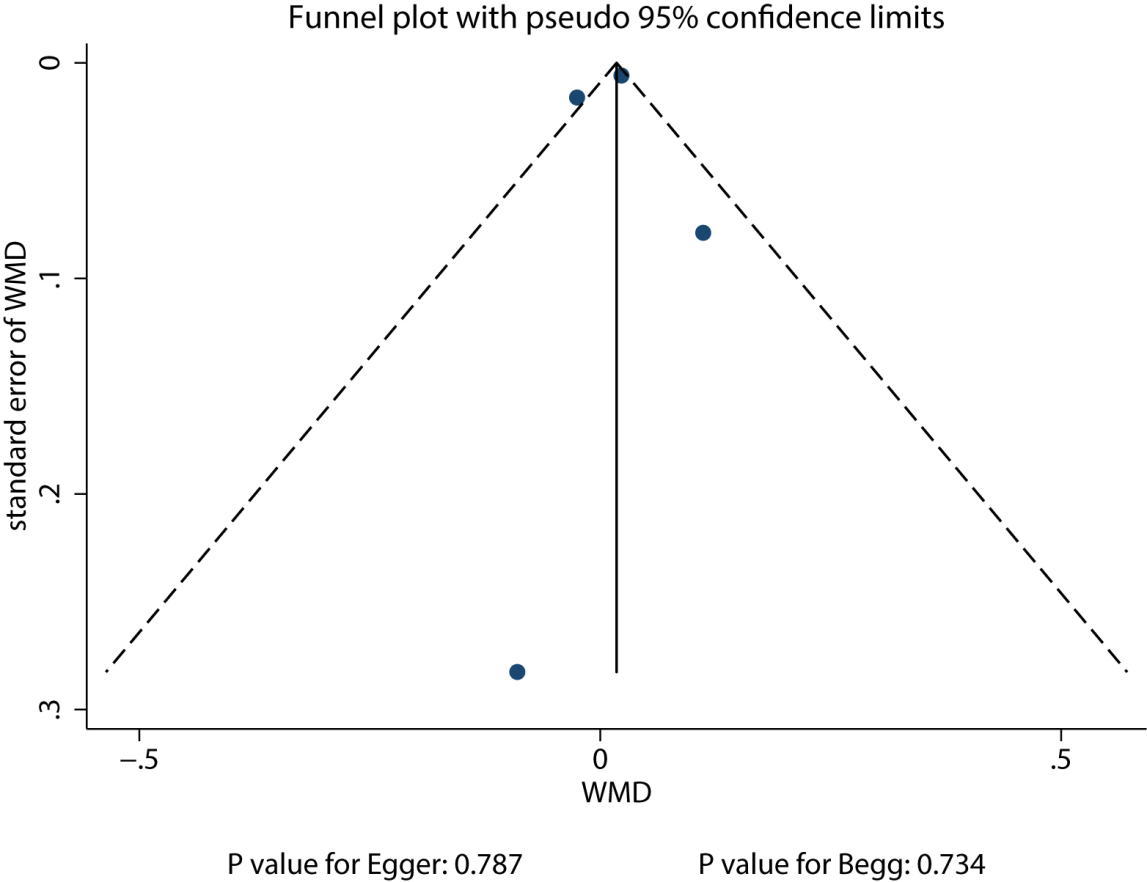


Figure S3. Funnel plot for BMD at Greater trochanter of the femur


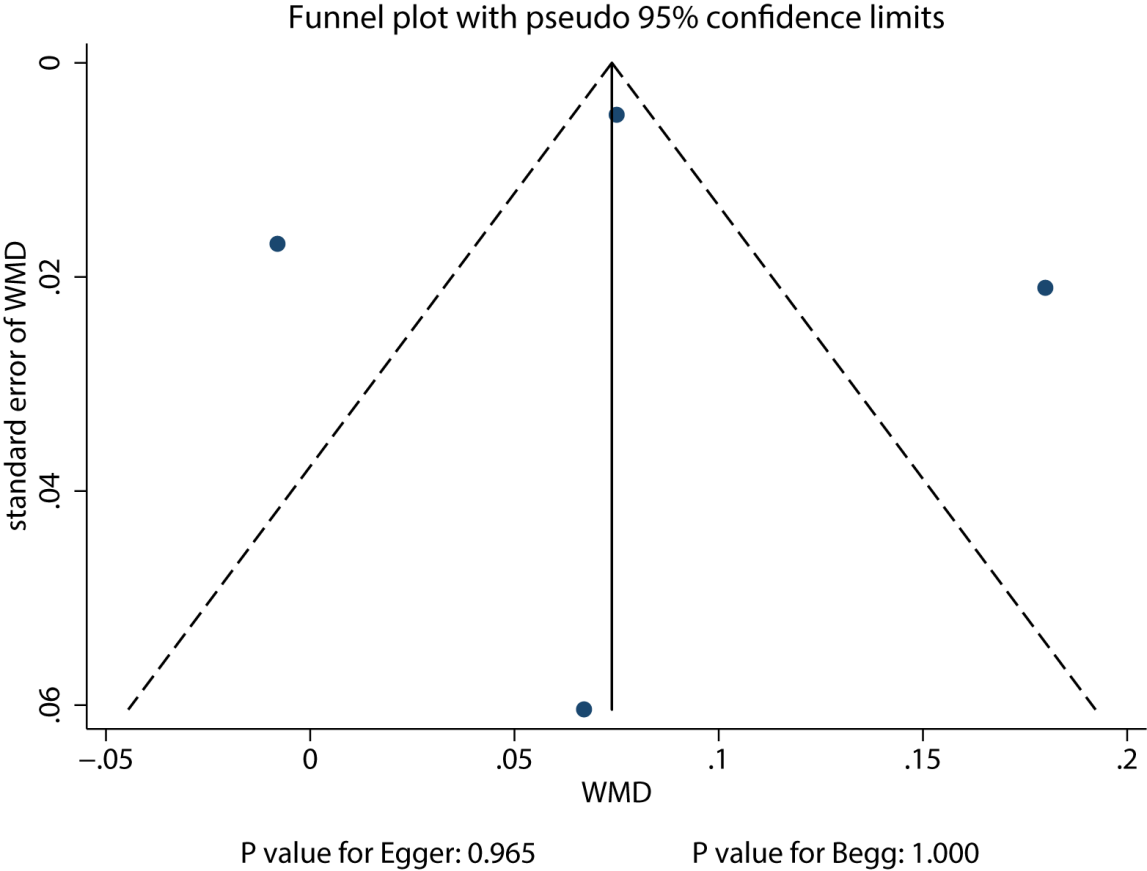


Figure S4. Funnel plot for BMD at Ward’s triangle area


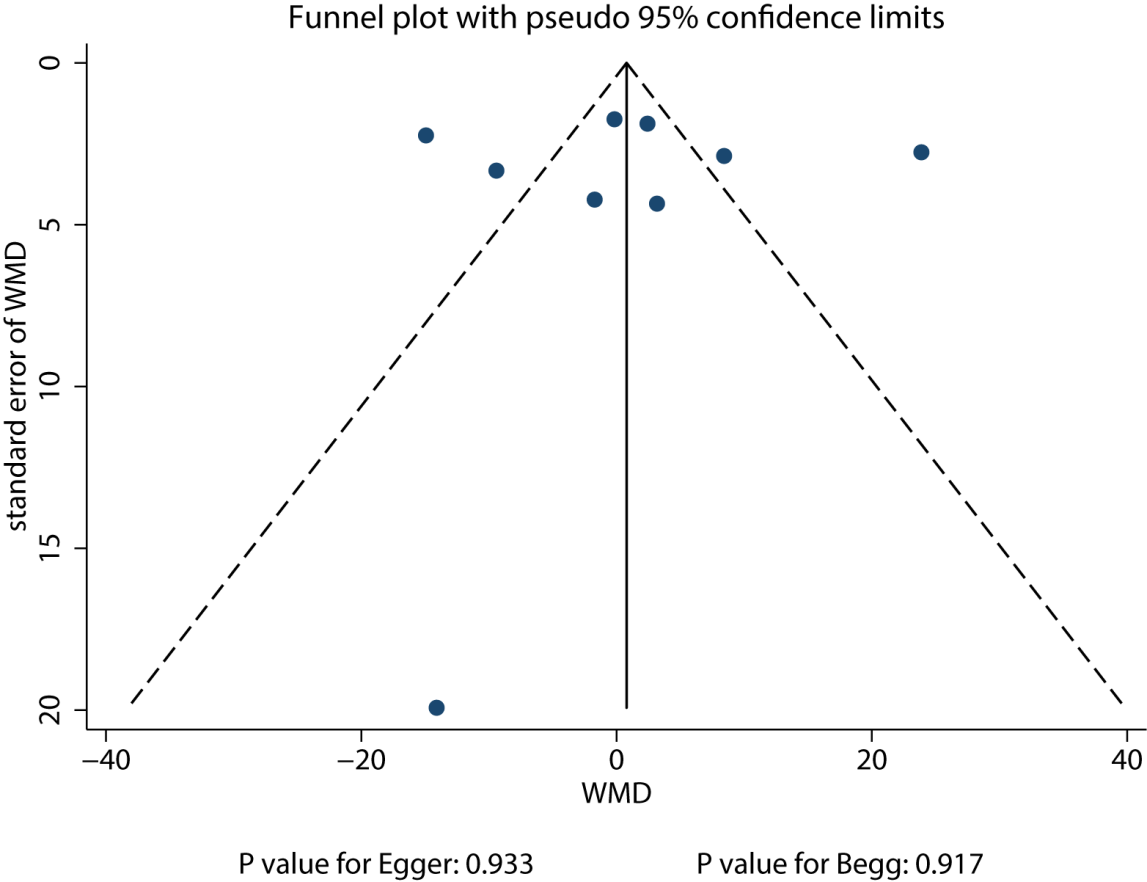


Figure S5. Funnel plot for ALP


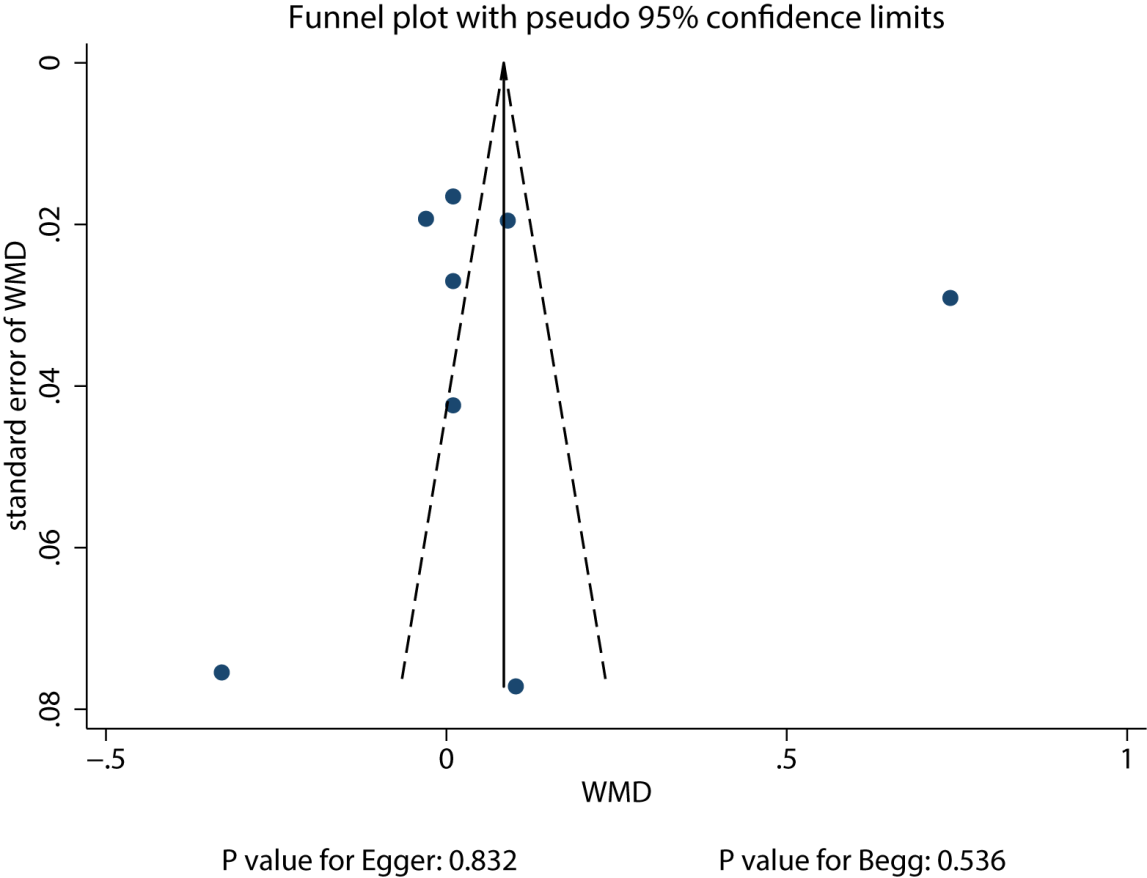


Figure S6. Funnel plot for serum calcium


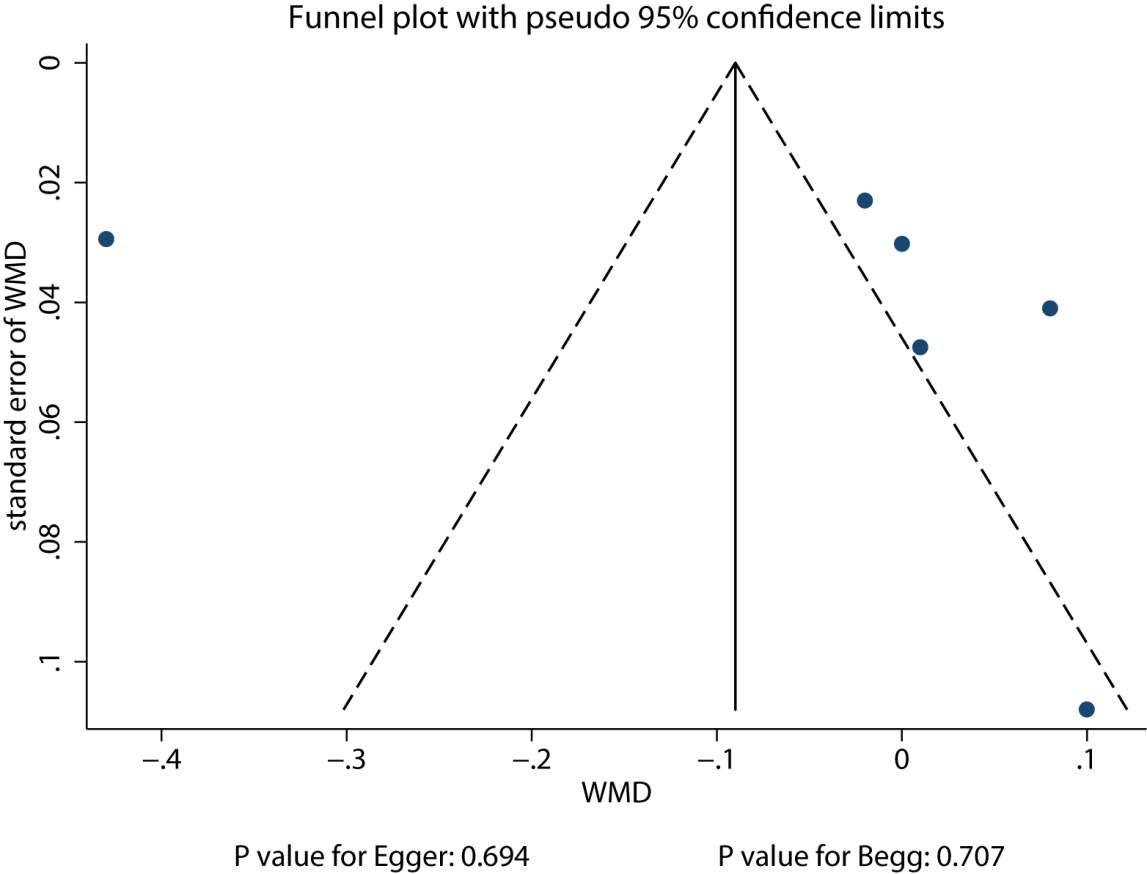


Figure S7. Funnel plot for serum P
